# Supplementary material for: Selenium catalysis enables negative feedback organic oscillators
Source: Nat Commun. 2024 Apr 17;15:3316. doi: 10.1038/s41467-024-47714-6 (PMC11024130; doi:10.1038/s41467-024-47714-6)
Supplement: Supplementary file 1 — Supplementary Information [file 41467_2024_47714_MOESM1_ESM.pdf]

Supplementary Information for

Selenium catalysis enables negative feedback organic oscillators

Xiuxiu Li, Polina Fomitskaia, Viktoryia A. Smaliak, Barbara S. Smith, Ekaterina V. Skorb, and Sergey N. Semenov*

*Correspondence to: sergey.semenov@weizmann.ac.il

Table of Contents

1. Materials and Methods 3

2. Synthesis 4

3. Flow experiments 8

3.1. Flow setup 8

3.2. Standard protocol for oscillations in flow 9

4. Batch kinetic experiments 10

4.1. ^{1}H NMR kinetics experiments with proinhibitors 3-5 10

4.2. Kinetics of autocatalysis 11

4.3 Additional studies of kinetics of oxidation of cysteamine by tBuOOH 13

4.4 Additional studies of kinetics of oxidation of thiocholine by tBuOOH 16

5. Derivation of the rate equation for the oxidation of thiols catalyzed by 4-carboxyselenophenol 17

6. Modeling 23

7. NMR spectra 28

8. Supplementary references 34

1. Materials and Methods

Unless otherwise noted, all chemicals were purchased from Sigma-Aldrich, Acros Organics, Alfa Aesar, and Merck. All solvents were purchased from Sigma-Aldrich and Acros Organics. D_2O was purchased from Tzamal d-chem; all other NMR solvents were purchased from Cambridge Isotope Laboratories. All chemicals, including solvents, were used without further purification. LC-MS grade water was used in all kinetic experiments.

NMR spectra were measured on a Bruker AVANCE III-300 spectrometer at 300 MHz for ^{1}H, at 73.7 MHz for $^{13}C\{1H\}$, on a Bruker AVANCE III-400 spectrometer at 400 MHz for ^{1}H, at 100.6 MHz for $^{13}C\{1H\}$, on a Bruker AVANCE III HD-500 spectrometer at 500 MHz for ^{1}H, and at 125.8 MHz for $^{13}C\{1H\}$. Chemical shifts for ^{1}H and ^{13}C are given in ppm relative to TMS, and for ^{77}Se relative to $Se(Me)_2$. ^{1}H and ^{13}C spectra were calibrated using a residual solvent peak as an internal reference (D_2O ^{1}H NMR: $\delta = 4.79$ ppm; $CDCl_3$ ^{1}H NMR: $\delta = 7.26$ ppm, ^{13}C NMR: $\delta = 77$ ppm; DMSO-d_6 ^{1}H NMR: $\delta = 2.50$ ppm, ^{13}C NMR: $\delta = 39.52$ ppm). Data for the ^{1}H NMR spectra were reported as follows: chemical shift (ppm), peak shape (s = singlet, d = doublet, t = triplet, q = quartet, p = pentet, m = multiplet, br = broad, dd = doublet of doublets), coupling constant (Hz), and integration.

High-Resolution Mass spectra were measured on a Waters Xevo G2-XS QTof mass spectrometer with an electrospray ionization (ESI) source spectrometer. All spectra were acquired in the mass range of 50–2000 m/z. The mass errors of the analyzed spectra are not more than 5.0 ppm.

The absorbance in the flow experiments was constantly monitored by a Cary 60 UV-VIS spectrometer, manufactured by Agilent Technologies. The flow cell used in these experiments was a hand-made cell. The reactants in the flow experiments were supplied by NEMESYS Low Pressure Syringe pumps produced by CETONI GmbH.

2. Synthesis

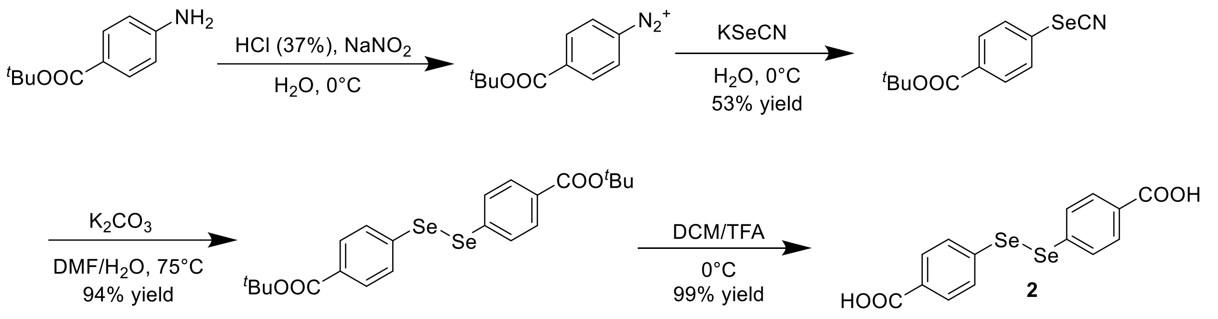

Supplementary Figure 1. The procedure for the synthesis of catalyst **2**

Tert-butyl 4-selenocyanatobenzoate was prepared following Nakamura et al.[1] First, 37% HCl (5.625 mL) was added dropwise to a 250 mL flask charged with tert-butyl 4-aminobenzoate (7,5 g, 0.039 mol) and water (50 mL) in an ice-water bath. After 5 min of stirring at 0°C, a $NaNO_2$ (2.22 g, 0.039 mol) solution in H_2O (5 mL) was slowly added dropwise. The reaction was stirred for 10 more minutes. A freshly prepared KSeCN (4.66 g, 0.039 mol) solution in H_2O (5 mL) was added dropwise, keeping the temperature below 5°C. The reaction was stirred at this temperature for 30 min, and then at room temperature for 3 h. The mixture was extracted with Et_2O, washed with water (2x20 mL) and brine (1x20mL), then dried with Na_2SO_4 and concentrated under reduced pressure. The crude product was purified by column chromatography eluting with hexane/EA (v/v = 40/1) in 53% yield (5.83 g, 20.7 mmol).

***tert*-butyl 4-selenocyanatobenzoate**:

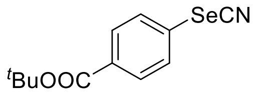

Red-brown solid (5.83 g, 20.7 mmol, 53% yield). ^{1}H NMR (300 MHz, $CDCl_3$) δ 8.00 (d, J = 8.5 Hz, 2H), 7.65 (d, J = 8.5 Hz, 2H), 1.60 (s, 9H).

Di-*tert*-butyl 4,4'-diselanediyldibenzoate was prepared using the oxidation procedure reported by Krief et al.[2] The solution of *tert*-butyl 4-selenocyanatobenzoate (0.45 g, 1.6 mmol) in DMF (4 mL) was mixed with the solution of K_2CO_3 (0.22 g, 1.6 mmol) in water (1 mL) and stirred for 2 h at 75°C. Next, the reaction mixture was quenched with $KHSO_4$ solution until a neutral pH was reached, extracted with DCM, washed with water (2x10mL), brine (2x10mL), and then dried with Na_2SO_4. Finally, the crude product was purified by column chromatography eluting with hexane/EA (v/v = 50/1) in 94% yield (0.38 g, 0.75 mmol).

Di-*tert*-butyl 4,4'-diselanediyldibenzoate:

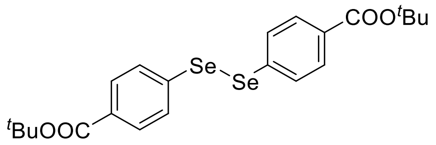

Yellow solid (0.38 g, 0.75 mmol, 94% yield). ^{1}H NMR (500 MHz, $CDCl_3$) δ 7.86 (d, *J* = 8.3 Hz, 4H), 7.62 (d, *J* = 8.3 Hz, 4H), 1.57 (s, 18H). ^{13}C NMR (126 MHz, CDCl3) δ 165.12, 135.85, 131.27, 130.13, 130.10, 81.24, 28.15 ppm. ^{77}Se NMR (95 MHz, $CDCl_3$) δ 446.79 ppm.

The solution of di-*tert*-butyl 4,4'-diselanediyldibenzoate (0.3 g, 0.59 mmol) was dissolved in 2 mL DCM, and TFA (2 mL) was added at 0°C. After 15 min of stirring at 0°C and 45 min at room temperature, the reaction mixture was evaporated until dryness, resulting in clean 4,4'-diselanediyldibenzoic acid almost in quantitative yield (236 mg, 0.59 mmol).

4,4'-diselanediyldibenzoic acid:

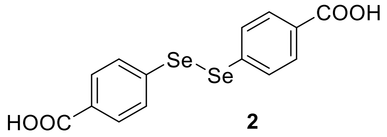

2

Pale yellow solid (236 mg, 0.59 mmol, 99% yield). ^{1}H NMR (500 MHz, DMSO-d_6) δ 13.02 (s, 1H), 7.87 (d, *J* = 9.9 Hz, 2H), 7.76 (d, *J* = 9.9 Hz, 2H). ^{13}C NMR (126 MHz, DMSO-d_6) δ 167.23, 136.13, 130.68, 130.47, 130.43 ppm. ^{77}Se NMR (95 MHz, DMSO-d_6) δ 433.72 ppm. HRMS (ESI) m/z: [M - H]$^-$ calcd. for $C_{14}H_9O_4Se_2$ 400.8831; found 400.8835.

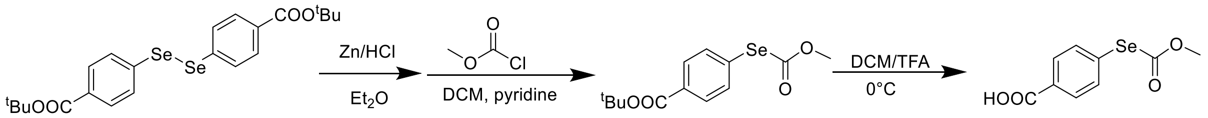

3

Supplementary Figure 2. The procedure for the synthesis of catalyst **3**

To the solution of di-*tert*-butyl 4,4'-diselanediyldibenzoate (0.197 g, 0.38 mmol) in Et_2O (3 mL) was added a scoop of powdered Zn, water (1 mL), and HCl 37% (2 mL). A mixture was closed with a septum, bubbled with Ar and left for 30 min. After the organic phase was decolorized, it was extracted with Et_2O (3x2 mL) into a flask charged with Na_2SO_4 under Ar flow.

To the solution of methyl chloroformate (59 μL, 0.76 mmol) in DCM (2 mL), the previous ether solution was added. Then, a pyridine (0.124 mL, 1.52 mmol) solution in DCM (1 mL)

was added dropwise and it was stirred for 2h. Next, the reaction mixture was diluted with DCM, washed with 1M HCl (1x10 mL), Na_2CO_3 (1x10mL), water (1x10 mL), and brine (1x10 mL), and then dried with Na_2SO_4. Finally, the resulting oil was purified using column chromatography eluting with hexane/EA (v/v = 20/1) in 92% yield (220 mg, 0.7 mmol).

The solution of *tert*-butyl 4-((methoxycarbonyl)selanyl)benzoate (0.22 g, 0.7 mmol) was dissolved in 3 mL DCM, and TFA (3 mL) was added at 0°C. After 15 min of stirring at 0°C and 45 min at room temperature, the reaction mixture was evaporated until dryness, resulting in clean 4-((methoxycarbonyl)selanyl)benzoic acid almost in quantitative yield (182 mg, 0.7 mmol).

4-((methoxycarbonyl)selanyl)benzoic acid:

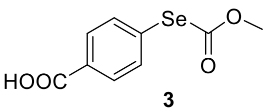

3

White solid, 92% overall yield in 3 steps (182 mg, 0.7 mmol). ^1H NMR (300 MHz, $CDCl_3$) δ 8.08 (d, J = 8.4 Hz, 2H), 7.75 (d, J = 8.4 Hz, 2H), 3.89 (s, 3H). ^{13}C NMR (126 MHz, $CDCl_3$) δ 171.70, 166.57, 135.37, 133.47, 131.05, 129.86, 55.37 ppm. ^{77}Se NMR (95 MHz, $CDCl_3$) δ 506.33 ppm. HRMS (ESI) m/z: $[M - H]^-$ calcd. for $C_9H_7O_4Se$ 258.9510; found 298.9504.

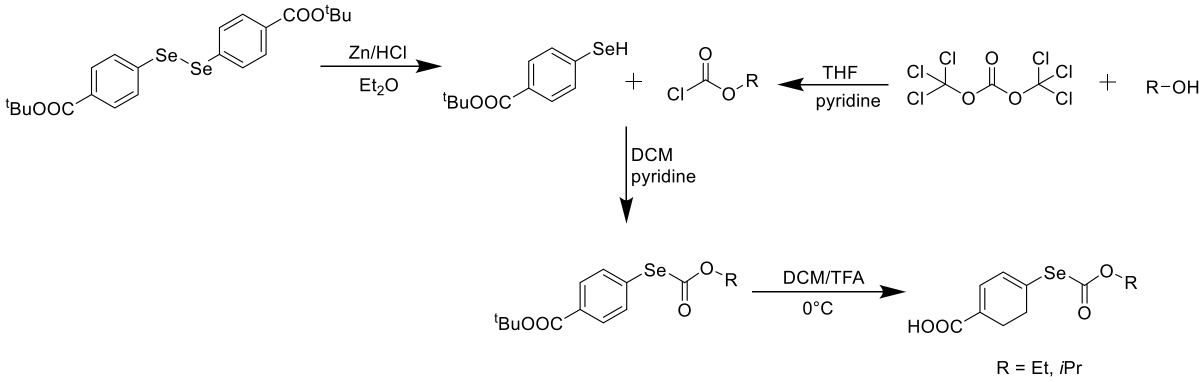

Supplementary Figure 3. The procedure for the synthesis of catalyst **4** and **5**

1. To the solution of di-*tert*-butyl 4,4'-diselanediyldibenzoate (0.197 g, 0.38 mmol) in Et_2O (3 mL) was added a scoop of powdered Zn, water (1 mL) and HCl 37% (2 mL). The mixture was closed with a septum, bubbled with Ar, and left for 30 min. After the organic phase was decolorized, it was extracted with Et_2O (3x2 mL) into a flask charged with Na_2SO_4 under Ar flow.

2. To a flask containing the solution of the corresponding alcohol (0.76 mmol) and triphosgene (0.113 g, 0.38 mmol) in dry THF (3 mL), a pyridine (0.061 mL, 0.76 mmol) solution in THF

(0.5 mL) was added. After having been stirred for 40 minutes, the resulting solution was directly used in the third step reaction.

3. To a flask with the product of step 2, the ether solution from step 1 was added. Then the pyridine (0.124 mL, 1.52 mmol) solution in DCM (1 mL) was added dropwise and stirred for 2h. Next, the reaction mixture was diluted with DCM, washed with 1M HCl (1x10 mL), Na_2CO_3 (1x10mL), water (1x10 mL), and brine (1x10 mL), and then dried with Na_2SO_4. Finally, the resulting oil was purified using column chromatography eluting with hexane/EA (v/v = 25/1 – 20/1).

4. The solution of the above compound was dissolved in 3 mL DCM, and TFA (3 mL) was added at 0°C. After 15 min of stirring at 0°C and 45 min at room temperature, the reaction mixture was evaporated until dryness, resulting in the desired compound, **4** or **5** almost in quantitative yield.

4-((ethoxycarbonyl)selanyl)benzoic acid:

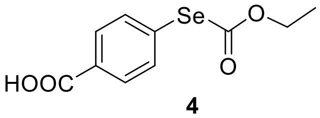

4

White solid, 88% overall yield in 4 steps (183 mg, 0.67 mmol). ^{1}H NMR (300 MHz, $CDCl_3$) δ 8.01 (d, *J* = 8.4 Hz, 2H), 7.68 (d, *J* = 8.4 Hz, 2H), 4.29 (q, *J* = 7.1 Hz, 2H), 1.27 (t, *J* = 7.1 Hz, 3H). ^{13}C NMR (126 MHz, $CDCl_3$) δ 170.86, 165.61, 135.36, 133.71, 131.00, 129.72, 65.19, 14.61 ppm. ^{77}Se NMR (95 MHz, $CDCl_3$) δ 513.21 ppm. HRMS (ESI) m/z: $[M - H]^-$ calcd. for $C_{10}H_9O_4Se$ 272.9666; found 272.9662.

4-((isopropoxycarbonyl)selanyl)benzoic acid:

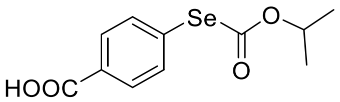

5

White solid, 82% overall yield in 4 steps (179 mg, 0.62 mmol). ^{1}H NMR (500 MHz, $CDCl_3$) δ 8.07 (d, *J* = 8.2 Hz, 2H), 7.74 (d, *J* = 8.2 Hz, 2H), 5.19 (hept, *J* = 6.2 Hz, 1H), 1.33 (d, *J* = 6.2 Hz, 6H). ^{13}C NMR (126 MHz, $CDCl_3$) δ 171.44, 164.30, 135.06, 133.75, 130.68, 129.33, 73.61, 21.92 ppm. ^{77}Se NMR (95 MHz, $CDCl_3$) δ 510.96 ppm. HRMS (ESI) m/z: $[M - H]^-$ calcd. for $C_{11}H_{11}O_4Se$ 286.9823; found 286.9818.

3 Flow experiments

3.1 Flow set-up

The flow set-up consisted of four main components: (i) syringe pumps, (ii) a micro continuously stirred tank reactor (CSTR), (iii) a microfluidic mixer (or two mixers), and (iv) a flow cell. We used NEMESYS Low Pressure Syringe pumps from CETONI in all our experiments.

Calibration of the flow cell. We used the calibration curves to convert the absorption to the concentration values and thus, to quantify the UV data from the oscillatory reactions in CSTR. To obtain the calibration curves, we mixed one volume of the mercaptoethanol solution of a corresponding concentration in Tris-buffer pH 7.7 with three volumes of the water/methanol (1.5/1) solution of Ellman's reagent (16.6 mM) also containing KH_2PO_4 (146 mM). Then, we set the absorbance to 0 for pure Tris-buffer, passed the resulting solution through our self-made flow cell and recorded the absorbance of the solutions. The whole sequence of preparing the solution and measuring the absorbance was repeated three times for each concentration of mercaptoethanol. The mean values were plotted and fitted with the following function:

$Y = a(1 - b^X)$,

where Y is the absorbance, X is the concentration of thiols, and a, b are the parameters.

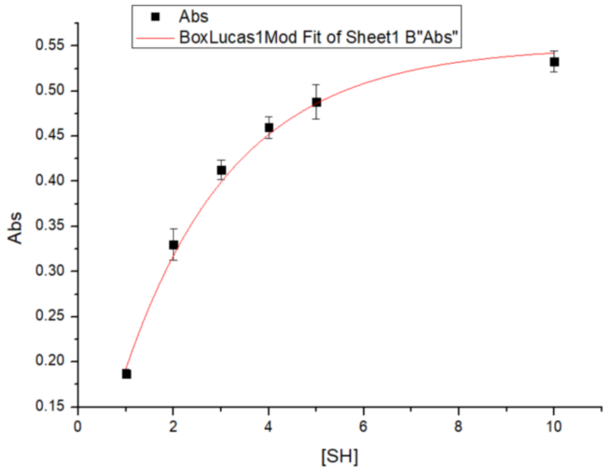

Supplementary Figure 4. Calibration curves of the thiol concentration in a home-made flow cell using Ellman's reagents. The background was corrected by subtracting the absorbance of Tris-buffer. Nonlinear fitting of the asymptotic function $Y = a(1 - b^X)$ was performed using Origin 9. The fitted parameters are as follows: $a = 0.54941 \pm 0.01215$ and

b = 0.649 ± 0.0.01218. The error bars represent the standard deviations from three independent measurements.

3.2 The standard protocol for oscillations in flow

Four syringes were filled with the required solutions as described below:

- 163 mg of thiouronium salt **8** and 64 mg of *t*BuOOH were dissolved in 2.5 mL of HPLC-grade water; next, this solution was transferred into a syringe. An additional 0.5 mL of water was used to wash the vial in which the solution was prepared, and the volume in the solution in the syringe was precisely filled to 3 mL. The same method was used to control the total volume of the solutions when filling the other syringes. The final concentration of **8** in the syringe was 168 mM, and the final concentration of *t*BuOOH in the syringe was 218 mM.

- Different selenium catalysts in various amounts were dissolved in 20 μL DMF and 2 mL water. The solution was transferred to a syringe and its volume was adjusted to 3 mL. The final concentrations in the syringe were 0.6 mM for **2**, 1.5 mM for **3**, 3.0 mM for **4**, and 6.0 mM for **5**.

- Tris-buffer (3M) was used to prepare the solution for the third syringe. The buffer was prepared to pH 7.7 after a threefold dilution to a concentration of 1 M. Thus, to obtain 100 mL of a buffer solution, 8.395 g (0.0693 mol) of anhydrous Tris-amine and 36.311 g (0.2304 mol) of anhydrous Tris-HCl were placed in a 100 mL volumetric flask and filled with HPLC grade water up to 100 mL. This Tris-buffer was used to prepare a 3 mL solution of 203 mg of cystamine dihydrochloride salt in a third syringe. The final concentration of cystamine in the syringe was 300 mM for all experiments.

- To fill the last syringe, two solutions were prepared separately. First, 10 mL of HPLC grade methanol was used to dissolve 164 mg (414 mmol) of 5,5'-dithiobis-(2-nitrobenzoic acid) (Ellman's reagent) and 15 mL of HPLC grade water was used to dissolve 0.5 g of KH_2PO_4. Those two solutions were combined and a 25 mL glass syringe was filled with solution through a 0.22 μm syringe filter. The final concentration of Ellman's reagent in the syringe was 16.6 mM

All 5 mL syringes (with 3 mL of solution each) were installed in the syringe pump system and connected to the CSTR using 0.5 mm internal diameter PTFE tubing. The CSTR outlet tubing was connected to the microfluidic mixer where the content of CSTR was mixed with Ellman's

reagent. For experiments with catalysts **3-5**, the flow rate for syringes 1-3 was 640 µL/h. For the experiment with catalysts **2**, the flow rate for syringes 1 and 3 was 640 µL/h, whereas the flow rate for syringe 2 was 400 µL/h. In all experiments, the flow of the solution of Ellman's reagent was three times higher than the flow from CSTR. After mixing, the flow passed through the flow cell, where absorbance at 412 nm was measured.

4. Batch kinetic experiments

4.1 *^{1}H NMR kinetics experiments with proinhibitors 3-5*

The kinetics of the interaction between proinhibitors **3-5** and cysteamine was monitored by ^{1}H NMR. In these experiments, we prepared a solution of a proinhibitor (**3**, **4**, or **5**) in 1 M Tris buffer pH 7.5, D_2O. The amounts of **3-5** were calculated to produce a 5 mM solution after dilution to a final volume of 600 µL. Next, we prepared the solution of cysteamine hydrochloride (0.68 mg, 10 mM after dilution to 600 µL) in the same buffer. The solutions were mixed, and ^{1}H NMR spectra were recorded every minute. The concentrations of **3-5** during the reactions (Figure 3b main text) were calculated by integrating signals in the aromatic region, then plotted and fitted with Supplementary equation 1 for a second order reaction with the non-equivalent initial concentrations of the reactants:

Supplementary equation 1
$$[A] = (B_0 - A_0) A_0 \frac{e^{-(B_0 - A_0)k(t+t_0)}}{B_0 - A_0 e^{-(B_0 - A_0)k(t+t_0)}}$$

where A_0 is the initial concentration of **3-5**, B_0 is the initial concentration of cysteamine, k is the second order rate constant, and t_0 is the correction factor for uncertainty in the starting time of the reaction. During fitting, we fixed A_0 at 0.005 and B_0 at 0.01. Experiments were conducted in triplicate. The experimental and fitting results are summarized in Supplementary Figure 5-7.

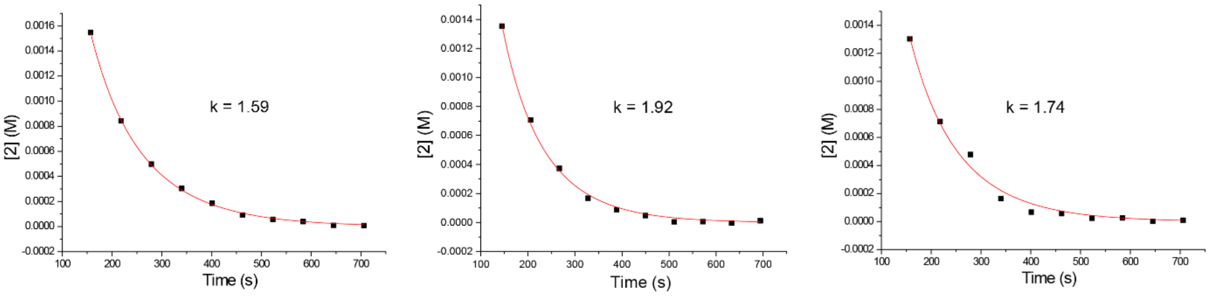

Supplementary Figure 5. Kinetics of the reaction between **3** and cysteamine. Parameters obtained by fitting with Supplementary equation 1: left (k = 1.59 $s^{-1}M^{-1}$; t_0 = -63 s), middle (k = 1.92 $s^{-1}M^{-1}$; t_0 = -57 s), and left (k = 1.74 $s^{-1}M^{-1}$; t_0 = -57 s).

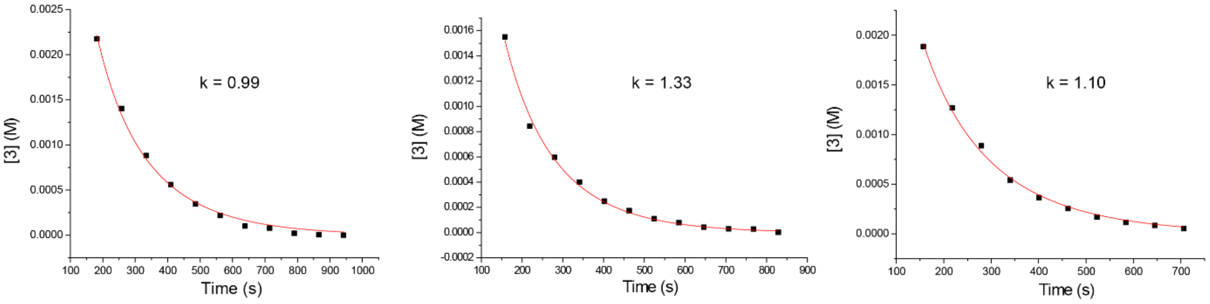

Supplementary Figure 6. Kinetics of the reaction between **4** and cysteamine. Parameters obtained by fitting with Supplementary equation 1: left (k = 0.99 $s^{-1}M^{-1}$; t_0 = -84 s), middle (k = 1.33 $s^{-1}M^{-1}$; t_0 = -43 s), and left (k = 1.10 $s^{-1}M^{-1}$; t_0 = -50 s).

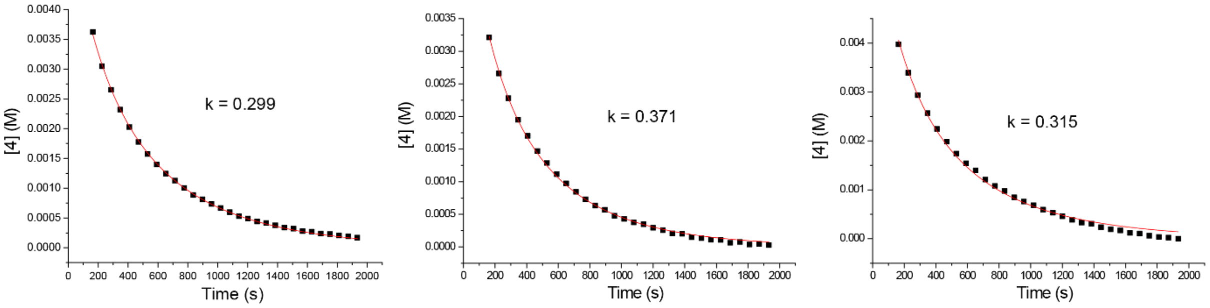

Supplementary Figure 7. Kinetics of the reaction between **5** and cysteamine. Parameters obtained by fitting with Supplementary equation 1: left (k = 0.299 $s^{-1}M^{-1}$; t_0 = -43 s), middle (k = 0.371 $s^{-1}M^{-1}$; t_0 = -32 s), and left (k = 0.315 $s^{-1}M^{-1}$; t_0 = -94 s).

4.2 *Kinetics of autocatalysis*

To estimate the rate constants (k_L, k_h, and k_{SS}) for the autocatalytic process, we measured the kinetics of the reaction between **8** (20 mM) and **9** (40 mM) in Tris buffer (1 M, pH 8) (Supplementary Figure 9). To follow the thiol concentrations in this reaction, we used the following experimental protocol: A freshly prepared solution of Ellman's reagent 2 mM in phosphate buffer solution (pH = 7.0; 200 mM) was used to fill about twenty UV-vis cuvettes with 2 mL of solution in each cuvette. Next, 10 μL aliquots of the reaction mixture were taken every few seconds and mixed with Ellman’s solution. The absorbance at 412 nm was measured by a UV-Vis spectrometer and converted to the total concentration of thiols.

This kinetics was fitted to the model defined by the following reactions (Supplementary Figure 8):

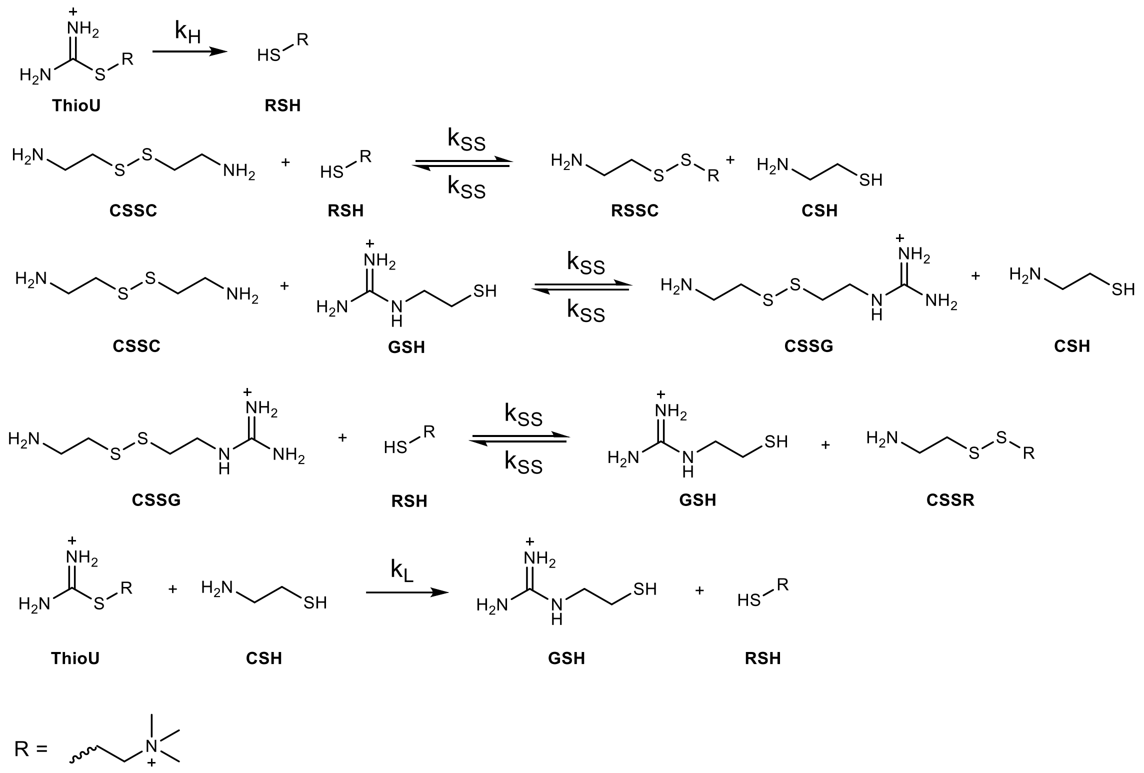

Supplementary Figure 8. Autocatalytic network from thiouronium salts and disulfides.

The modeling details of this autocatalyic network are similar to those we described previously.[3] In the current study, using literature estimates,[4] we fixed $k_{SS} = 5$ s^{-1}M^{-1} while optimizing k_h and k_L. The optimization results in $k_h = 0.00013 \pm 1{\cdot}10^{-5}$ s^{-1} and $k_L = 1.98 \pm 0.02$ s^{-1}M^{-1}. With these parameters, the model curve follows the experimental points very closely (Supplementary Figure 9).

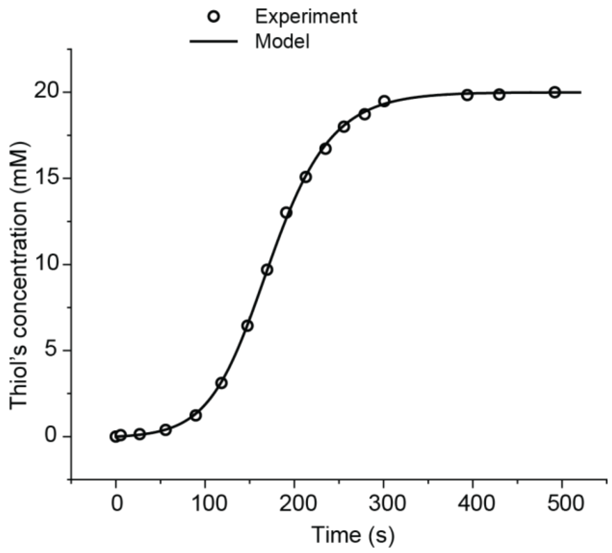

Supplementary Figure 9. Fitting the experimental data for the autocatalysis with the numerical model (see the numerical modeling section for details). Experimental conditions:

H_2O, 1 M Tris buffer pH 8, 25 °C; [**8**] = 20 mM; [**9**] = 40 mM. The model parameters obtained by fitting the experimental data are as follows: k_{SS} = 5 $s^{-1}M^{-1}$ (fixed), k_L = 1.98 ± 0.02 $s^{-1}M^{-1}$, and k_h = 0.00013 ± $1{\cdot}10^{-5}$ s^{-1}.

4.3 Additional studies of the kinetics of the oxidation of cysteamine by *t*BuOOH

Kinetics of the oxidation of cysteamine by tBuOOH without selenium derivatives

Generally, the solution of cysteamine in Tris buffer pH 7.5 was mixed with the solution of *t*BuOOH. The initial cysteamine concentration was in the range 0.5-10 mM and the initial *t*BuOOH concentration was in the range 3-39 mM. To follow the concentration of cysteamine in this reaction, we used the following experimental protocol: A freshly prepared solution of Ellman's reagent 2 mM in phosphate buffer solution (pH = 7.0; 200 mM) was used to fill UV-vis cuvettes with 2 mL of solution in each cuvette. Next, 20 µL aliquots of the reaction mixture were taken every few seconds and mixed with Ellman’s solution. The absorbance at 412 nm was measured by a UV-Vis spectrometer and converted to the total concentration of thiols.

To obtain the required plots, the absorbance data were analyzed as follows: First, the absorbance data were converted to cysteamine concentrations in the reaction mixture using an extinction coefficient of 14150 M^{-1} cm^{-1} and a dilution factor of 101 (20 µL aliquot vs 2020 µL of Ellman’s solution plus aliquot). Next, the concentration was plotted against time, and the initial linear regions identified and fitted with a linear function. The slopes of these lines represent the initial reaction rates. Measurements for each reaction rate point are repeated three times, and average values were used to make the required curves and to determine the reaction orders and the rate constants.

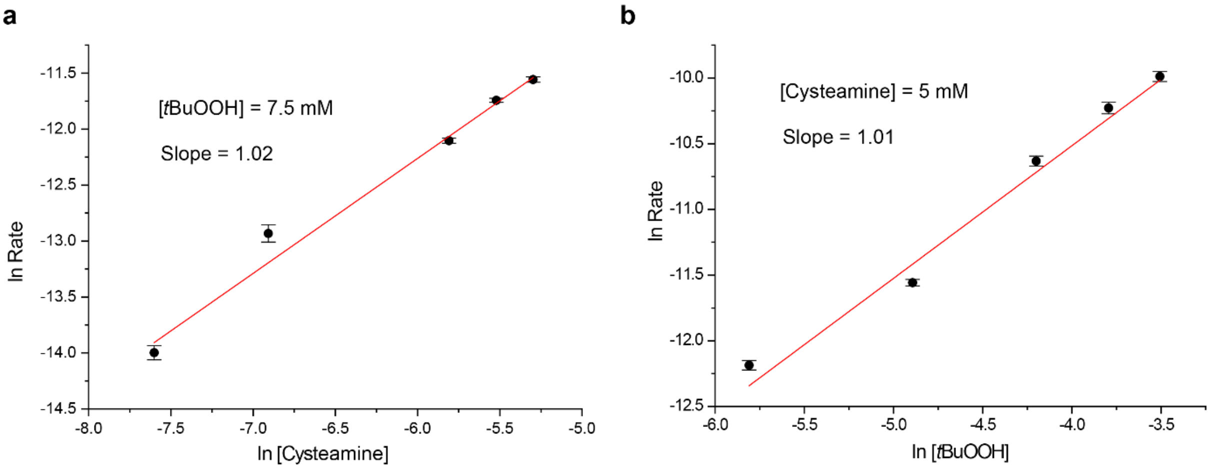

Supplementary Figure 10. Determination of the rate order of cysteamine and *t*BuOOH. a) Determination of the rate order of cysteamine. Reaction conditions: Tris buffer, pH 7.5, 1M, 25 °C, [*t*BuOOH] = 7.5 mM, [cysteamine] = 0.5 – 5 mM. b) Determination of the rate order of *t*BuOOH. Reaction conditions: Tris buffer, pH 7.5, 1M, 25 °C, [cysteamine] = 5 mM, [*t*BuOOH] = 3 - 30 mM. The error bars represent the standard deviations from three independent measurements.

As shown in Supplementary Figure 10, the oxidation of cysteamine by *t*BuOOH is the first order in cysteamine and the first order in *t*BuOOH. Therefore, the rate of this reaction can be described by the equation:

Supplementary equation 2
$$\frac{d[tBuOOH]}{dt} = \frac{1}{2}\frac{[Cys]}{dt} = -k[Cys][tBuOOH]$$

For future numerical modeling of the oscillations, we estimated the value for this rate constant at pH 7.5 in Tris buffer 1M in the presence of cystamine. The average value of the rate constant from these two series of experiments (Supplementary Figure 10) is 0.142 ± 0.008 $M^{-1}s^{-1}$.

Kinetics of the oxidation of cysteamine by tBuOOH catalyzed by selenium derivatives

We used a protocol identical to the one described in the previous section, but with the addition of the required amount of diselenide catalyst **2**. The concentration of **2** was converted to the concentration of selenium as [Se] = 2·[**2**]. Importantly, to analyze only the catalytic reaction, we subtracted the non-catalytic rate. Therefore, for each point of the se-catalyzed reaction rate, six measurements were made: three with catalyst **2** and three without it. Standard deviations were calculated as the sum of standard deviations for the three experiments with **2** and for three experiments without **2**.

Most data for the kinetics of the oxidation of cysteamine by *t*BuOOH catalyzed by selenium derivatives at pH 7.5 are presented in Figure 6 in the main text. Nevertheless, Michaelis-Menten plots for [Se] = 0.5 and 1 mM were omitted for clarity as well because of the somewhat unreliable fitting results, which most likely resulted from the difficulty in reliably measuring the initial reaction rates. The full set of Michaelis-Menten plots with variable amounts of **2** is shown in Supplementary Figure 11.

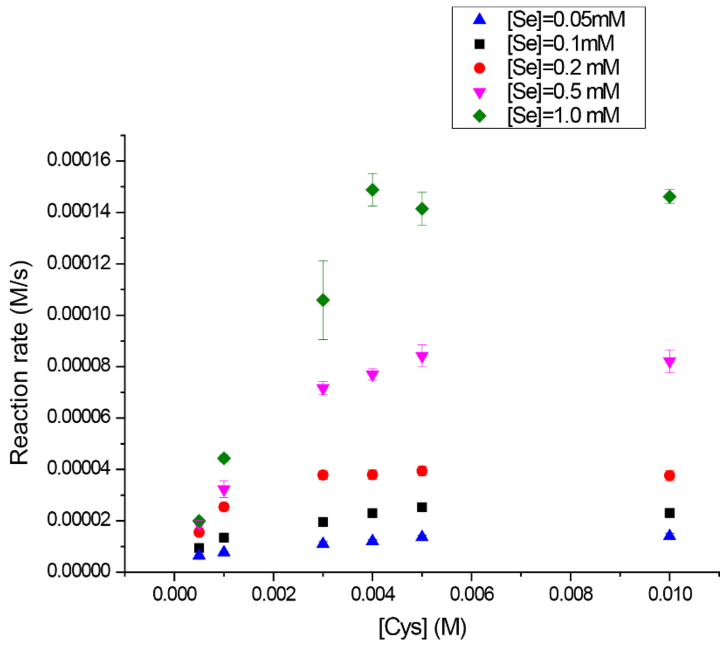

Supplementary Figure 11. Michaelis-Menten plots for the reaction between cysteamine and *t*BuOOH catalyzed by **2**. Reaction conditions: Tris buffer, pH 7.5, 1M, 25 °C, [*t*BuOOH] = 7.5 mM, [cysteamine] = 0.5 – 10 mM. The selenium concentrations (2·[**2**]) for each series are shown in the plot. The error bars represent the standard deviations from three independent measurements.

Comparison of the kinetics of the oxidation of cysteamine by tBuOOH at pH 7.5 and 7.7

Supplementary Table 1. Comparison of the reaction rates for the oxidation of cysteamine by *t*BuOOH at pH 7.5 and 7.7. Reaction conditions: Tris buffer, pH 7.5 or 7.7, 1M, 25 °C, [*t*BuOOH] = 7.5 mM, [cysteamine] = 5 mM, with or without Se, 0.5 mM. Errors indicate the standard deviation from three independent measurements.

Reaction	Initial rate (M/s)
pH 7.5 without Se	0.010±0.001
pH 7.5 with Se	0.088±0.002
pH 7.7 without Se	0.011±0.001
pH 7.7 with Se	0.073±0.005
pH 7.5 with Se, only the catalytic rate	0.078±0.003
pH 7.7 with Se, only the catalytic rate	0.062±0.006

4.4 Additional studies of the kinetics of the oxidation of thiocholine by *t*BuOOH

Kinetics of the oxidation of thiocholine by tBuOOH without selenium derivatives

The kinetics of this reaction was studied using the same protocols as in the oxidation of cysteamine. The results of the studies are summarized in Supplementary Figure 12.

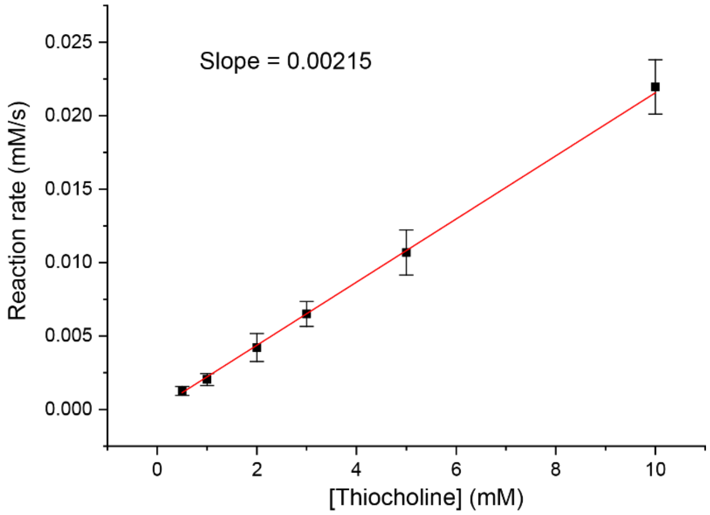

Supplementary Figure 12. Determination of the second order rate constant for the reaction between thiocholine and *t*BuOOH. Reaction conditions: Tris buffer, pH 7.5, 1M, 25 °C, [*t*BuOOH] = 7.5 mM, [Thiocholine] = 0.5 – 10 mM. The error bars represent the standard deviations from three independent measurements.

As shown in Supplementary equation 2, the slope of the curve from Supplementary Figure 12 corresponds to 2·*k*[*t*BuOOH]; therefore, $k = 0.143 \pm 0.003$ $M^{-1}s^{-1}$. We noted that the value of 0.143 $M^{-1}s^{-1}$ is an estimate; the actual values for rate constants under the conditions of the oscillatory experiments might differ significantly.

5. Derivation of the rate equation for the oxidation of thiols catalyzed by 4-carboxyselenophenol

Stoichiometric reaction

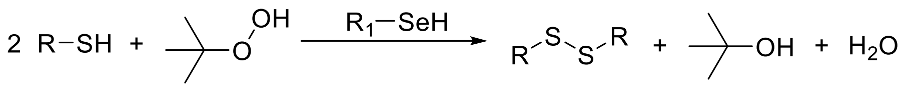

Supplementary Figure 13. Stoichiometric reaction between thiols and *tert*-butyl hydroperoxide.

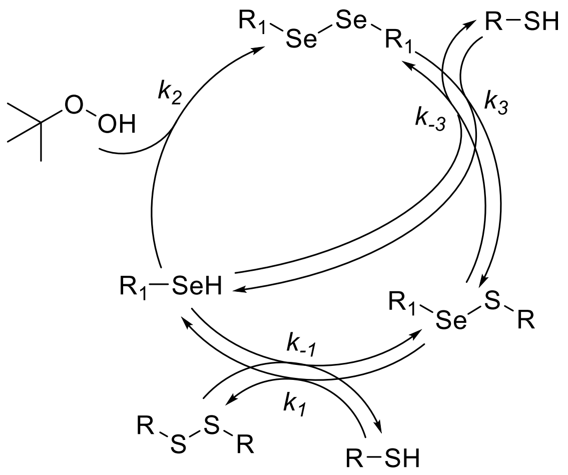

Supplementary Figure 14. Proposed mechanism for the catalytic cycle in the oxidation of thiols by *tert*-butyl hydroperoxide catalyzed by 4-carboxyselenophenol.

The proposed mechanism for catalysis results in a set of rate and mass-balance equations. The most relevant of them are listed below:

Supplementary equation 3 $$[R_1SeH]+2[R_1SeSeR_1]+[R_1SeSR]=Se_0$$

where Se_0 is the total amount of selenium introduced in the reaction mixture in the form of R_1SeSeR_1.

Supplementary equation 4 $$\frac{d[OOH]}{dt}=-k_2[R_1SeH][tBuOOH]$$

This equation already includes three approximation/assumptions. First, we assumed that the oxidation of selenols to diselenides with peroxides proceeds in two steps with the intermediate formation of R_1SeOH species. Second, we assumed that the formation of R_1SeOH from R_1SeH and tBuOOH is the rate limiting step in this reaction, which is the first order in R_1SeH and *t*BuOOH. Third, we neglected the reaction of R_1SeOH with RSH leading to R_1SeSR.

Supplementary equation 5

$$\frac{d[R_1SeH]}{dt} = k_3[R_1SeSeR_1] - k_{-3}[R_1SeH][R_1SeSR] + k_1[R_1SeSR][RSH]$$
$$-k_{-1}[R_1SeH][RSSR] - 2k_2[R_1SeH][tBuOOH]$$

Similar rate equations can be written for other species in the system, but the resulting system of nonlinear ODEs will not be tractable analytically.

To simplify the problem, we looked for possibilities to use a pseudo-equilibrium and steady-state approximations. It has been shown that the exchange between diselenides and thiols can be very fast.[5] Therefore, the exchange described by rate constants k_3 and k_{-3} could be a good candidate for applying pseudo-equilibrium approximation.

To study this equilibrium, we used ^{1}H NMR spectroscopy of the mixtures of 4,4'-diselanediyldibenzoic acid (**2**) and cysteamine. The composition of this mixture is defined by two equilibria:

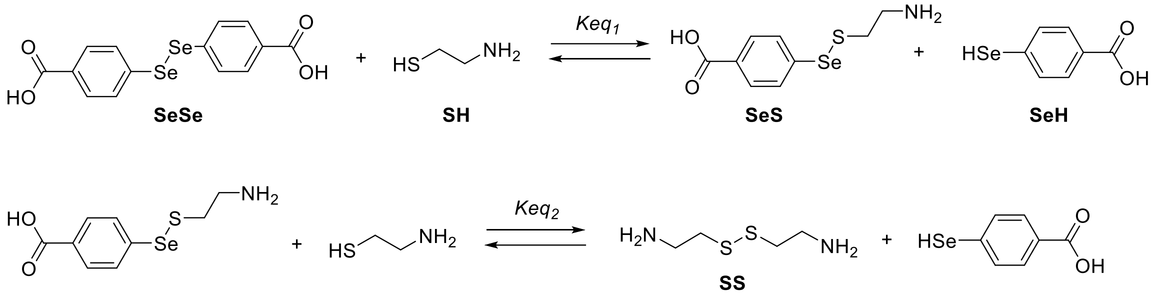

Supplementary Figure 15. Equilibria in the mixture of 4,4'-diselanediyldibenzoic acid and cysteamine.

We tested three mixtures in Tris buffer, 1 M, pH 7.5: **2** (10 mM), cysteamine (5 mM); **2** (10 mM), cysteamine (10 mM); **2** (10 mM), cysteamine (20 mM). ^{1}H NMRs of the resulting equilibrated mixtures are shown below:

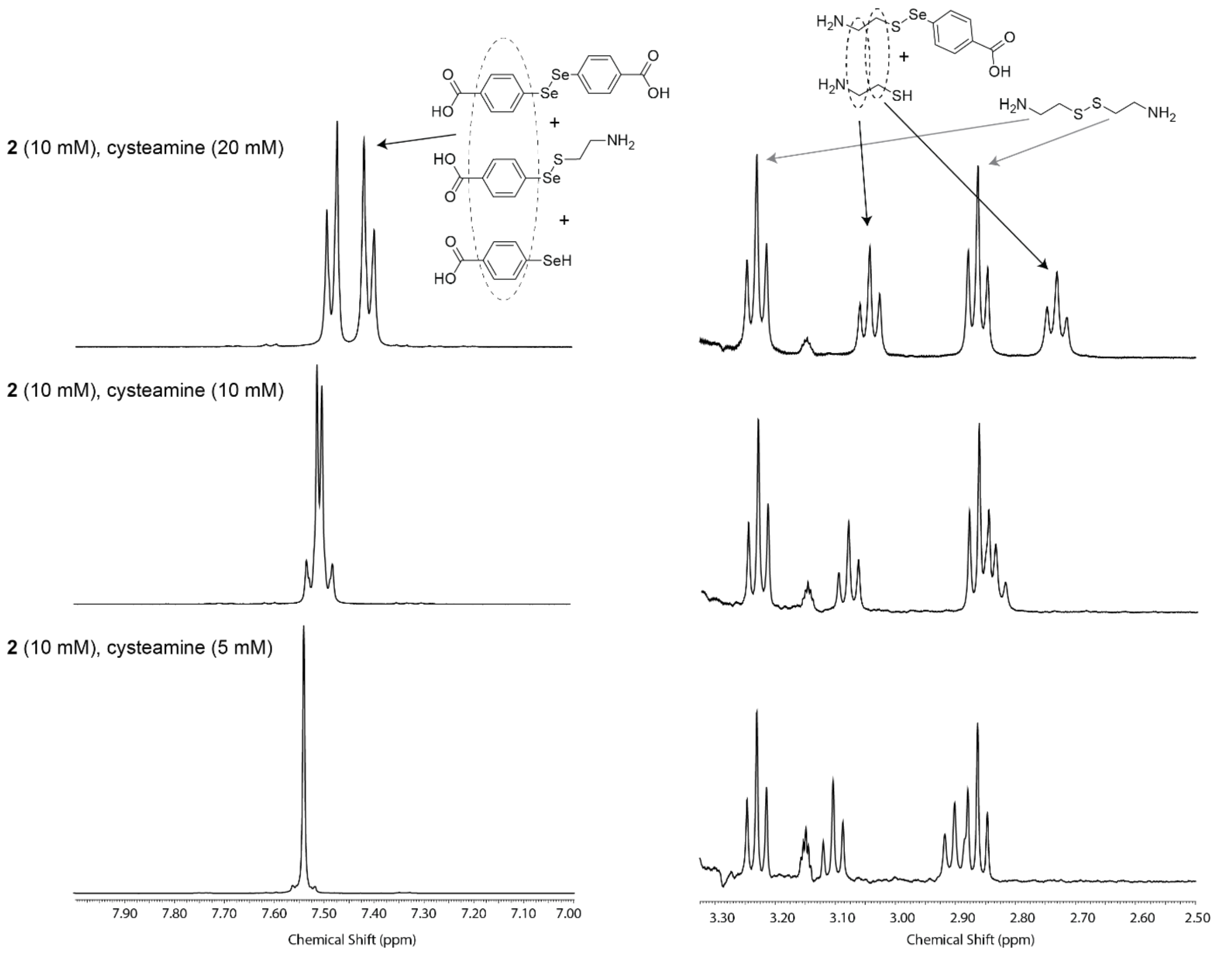

Supplementary Figure 16. ^{1}H NMR of different mixtures of **2** and cysteamine (500 MHz, D_2O).

Aromatic protons show only one signal that changes its position and shape, depending on the amount of cysteamine added. This behavior indicates that three derivatives (SeH, SeSe, and SeS) of 4-carboxyselenophenole participate in an equilibrium reaction or reactions that are fast in a NMR time scale. Methylene protons of cysteamine (SH) form two groups of signals: two triplets from cystamine (disulfide of cysteamine, SS) and two triplets that change their position, depending on the amount of cysteamine added. The moving triplets are likely to belong to the mixture of cysteamine (SH) and mixed selenosulfide (SeS). Because cystamine (SS), which has individual and stable signals, participates only in the second equilibrium, whereas all other compounds participate in the first equilibrium, we can conclude that the first equilibrium is fast in an NMR time scale, whereas the second equilibrium is not. This information corroborates well with previous findings that indicate that the first equilibrium in the diselenide thiol exchange involves much faster reactions than the second one and involves a rate constant of the order of $1 \cdot 10^6$ $s^{-1}M^{-1}$.[5] Therefore, it seems reasonable to apply equilibrium approximation

for the first exchange in the context of the oxidation of thiols by peroxides. The oxidation takes seconds to minutes and this equilibrium is established instantly using this time scale.

Although the averaging of signals for all compounds except cystamine prevents direct calculation of K_{eq1} and K_{eq2}, some information on these constants can be obtained from equilibrium cystamine concentrations obtained from NMR integration. The information is summarized in Supplementary Table 2.

Starting composition	Cystamine concentration from integration
**1** (10 mM), cysteamine (5 mM)	1.5 mM
**1** (10 mM), cysteamine (10 mM)	3.3 mM
**1** (10 mM), cysteamine (20 mM)	6.3 mM

Supplementary Table 2. Cystamine concentration at equilibrium.

The composition of equilibrium mixture is defined by a set of five equations:

Supplementary equation 6 $$\frac{[SeS][SeH]}{[SeSe][SH]} = K_{eq1}$$

Supplementary equation 7 $$\frac{[SS][SeH]}{[SeS][SH]} = K_{eq2}$$

Supplementary equation 8 $$2[SeSe] + [SeS] + [SeH] = Se_0,$$

where Se_0 is the total amount of selenium introduced in the reaction mixture in the form of *SeSe*.

Supplementary equation 9 $$[SH] + [SeS] + 2[SS] = S_0,$$

where S_0 is the total amount of sulfur introduced in the reaction mixture in the form of *SH*.

Supplementary equation 10 $$S_0 - [SH] = [SeH]$$

This system has a definitive solution if K_{eq1}, K_{eq2}, Se_0, and S_0 are known, but solving this system manually is impractical. Therefore, we used the following Mathematica code to estimate the influence of K_{eq1}, K_{eq2} on the equilibrium concentration of cystamine (variable ***v***):

Clear [*x*, *y*, *z*, *w*, *v*, *a*, *b*, *k*, *n*];

$a = 20$;

$b = 20$;

$k = 8$;

$n = 8$;

N[Solve[{*b* - *y* == *x*, 2**z* + *w* + *x* == *a*, *y* + *w* + 2**v* == b, *w***x*/(*z***y*) == *k*, *v***x*/(*w***y*) == *n*}, {*x*, *y*, *z*, *w*, *v*}]]

Using literature data showing that the equilibrium constants for the first and the second exchange are likely to be similar,[5] we can estimate that these equilibrium constants in our system are in the order of 10. Specifically, in the example with Se_0 = 20 mM (10 mM of SeSe) and S_0 = 20 mM, $K_{eq1} = K_{eq2} = 8$ results in an equilibrium concentration of cystamine of 6.36 mM, which is close to the experimental results of 6.3 mM (Supplementary Table 2).

Applying equilibrium approximations allows us to simplify Supplementary equation 1 and 3 as follows:

Supplementary equation 11

$$[R_1SeH]+2[R_1SeSeR_1]+[R_1SeSR]=[R_1SeH]+[R_1SeSR]+2\frac{[R_1SeSR][R_1SeH]}{K_{eq1}[RSH]}=Se_0$$

This equation can be transformed to:

Supplementary equation 12
$$[R_1SeSR]=\frac{K_{eq1}[RSH]Se_0-K_{eq1}[RSH][R_1SeH]}{K_{eq1}[RSH]+2[R_1SeH]}$$

Considering that K_{eq1} is likely to be higher than 1 and that in most experiments $[RSH] >> [R_1SeH]$, we can write:

Supplementary equation 13
$$[R_1SeSR]=\frac{K_{eq1}[RSH]Se_0-K_{eq1}[RSH][R_1SeH]}{K_{eq1}[RSH]+2[R_1SeH]}\approx Se_0-[R_1SeH]$$

Equilibrium approximation allows us to simplify Supplementary equation 3 to:

Supplementary equation 14

$$\frac{d[R_1SeH]}{dt}=k_1[R_1SeSR][RSH]-k_{-1}[R_1SeH][RSSR]-k_2[R_1SeH][tBuOOH]$$

because the sum of the first two terms in Supplementary equation 3 is zero at equilibrium.

The next major step in analyzing the kinetics of this system is to apply steady-state approximation for the central catalytic species in this reaction, which is *[R₁SeH]:*

Supplementary equation 15

$$\frac{d[R_1SeH]}{dt} = k_1[R_1SeSR][RSH] - k_{-1}[R_1SeH][RSSR] - 2k_2[R_1SeH][tBuOOH] = 0$$

Considering Supplementary equation 11, we can write:

Supplementary equation 16

$$k_1(Se_0 - [R_1SeH])[RSH] - k_{-1}[R_1SeH][RSSR] - 2k_2[R_1SeH][tBuOOH] = 0$$

This equation can be transformed into:

Supplementary equation 17 $$[R_1SeH] = \frac{k_1Se_0[RSH]}{k_{-1}[RSSR] + 2k_2[tBuOOH] + k_1[RSH]}$$

By using Supplementary equation 15 in Supplementary equation 2, we obtain:

Supplementary equation 18 $$\frac{d[OOH]}{dt} = -\frac{k_2k_1Se_0[RSH][tBuOOH]}{k_{-1}[RSSR] + 2k_2[tBuOOH] + k_1[RSH]}$$

Using the stoichiometry of the reaction at steady state for Se-containing species, we can write:

Supplementary equation 19 $$\frac{d[RSH]}{dt} = 2\frac{d[OOH]}{dt}$$

Using Supplementary equation 17, we can write:

Supplementary equation 20 $$\frac{d[RSH]}{dt} = -\frac{2k_2k_1Se_0[RSH][tBuOOH]}{k_{-1}[RSSR] + 2k_2[tBuOOH] + k_1[RSH]}$$

After transforming Supplementary equation 18 to a Michaelis-Menten-like form, we obtain:

Supplementary equation 21 $$\frac{d[RSH]}{dt} = -\frac{2k_2Se_0[RSH][tBuOOH]}{k_{-1}/k_1[RSSR] + 2k_2/k_1[tBuOOH] + [RSH]}$$

We can rewrite this equation as:

Supplementary equation 22 $$\frac{d[RSH]}{dt} = -\frac{k_2^*Se_0[RSH][tBuOOH]}{k_{-1}/k_1[RSSR] + k_2^*/k_1[tBuOOH] + [RSH]}$$

Supplementary equation 22 was used for analyzing the oscillations.

6. Modeling

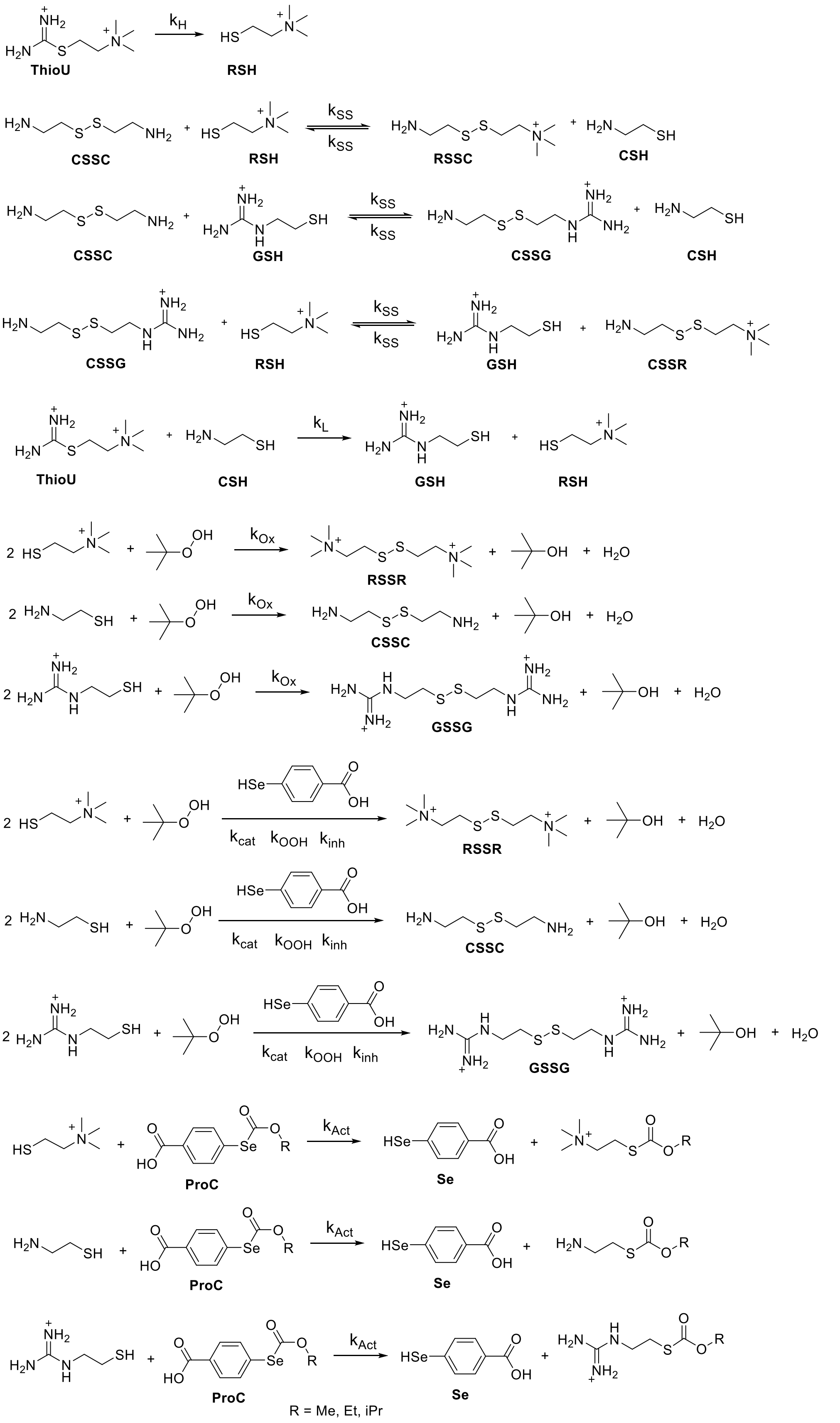

Supplementary Figure 17. The reactions used for the numerical simulation of the oscillations

in CSTR. The last three reactions were used only for oscillators with pre-catalysts **2**-**4**. The parameters involved here are as follows: k_H is the rate of the thiouronium salt hydrolysis; k_L is the rate of the ligation; k_{SS} is the rate of the disulfide exchange; k_{Ox} is the rate of the non-catalytic oxidation of thiols by *t*BuOOH; k_{cat}, k_{OOH}, and k_{inh} correspond to $k_2^*/2$, k_2^*/k_1, and $0.5k_{-1}/k_1$ from Supplementary equation 22, and k_{Act} is the second order rate constant for the release of 4-carboxyselenophenol. This k_{Act} constant differs between substrates **2**-**4**. All reactions except for the catalytic oxidation are assumed to follow the mass-action law. The rate law for the catalytic oxidation is described by equation 22. The rate law for flow supply and removal is described as the FvV × concentration.

The numerical model of the oscillation in CSTR (Supplementary Figure 17) was built using *COPASI* software.[6] Seven assumptions were made in this model. (1) All disulfide exchanges have the same rate constants. (2) The six disulfides were reduced to three "halves of disulfides". Therefore, the disulfide exchanges are also simplified to three exchange reactions. Accordingly, the total concentration of disulfides in the model was twofold higher than the experimental value. (3) The two stages of the thiol-assisted amination of the thiouronium salts were not separated in this model; instead, they were treated as one irreversible reaction. (4) Based on the experimental data for cysteamine and thiocholine, the non-catalytic oxidation of all three thiols was assumed to proceed with same rate constant, k_{Ox}. (5) The catalytic oxidation was assumed to follow Supplementary equation 22. (6) We assumed no difference between thiols in the reaction releasing 4-carboxyselenophenol from pre-catalysts **2**-**4**. (7) We neglected the rearrangement of thiocarbonate formed in the reaction of **2**-**4** with cysteamine into carbamate.

The behavior of the system was described by a set of ODEs (Supplementary Figure 18) and was solved numerically by using the *COPASI* program.

$$\frac{d([CSH]\cdot V_1)}{dt} = +V_1\cdot((Kss\cdot[CS]\cdot[HSR]-Kss\cdot[CSH]\cdot[SR]))$$
$$-V_1\cdot(KL\cdot[ThioU]\cdot[CSH])$$
$$-V_1\cdot(FvsV\cdot[CSH])$$
$$-2\cdot V_1\cdot(Kox\cdot[CSH]\cdot[TbOOH])$$
$$-V_1\cdot(Kact\cdot[ProC]\cdot[CSH])$$
$$-2\cdot V_1\cdot\left(\frac{[CSH]\cdot k1_{(Cat3a)}\cdot[Se]\cdot[TbOOH]}{k2_{(Cat3a)}\cdot([CS]+[GS]+[SR])+k3_{(Cat3a)}\cdot[TbOOH]+[CSH]+[HSR]+[GSH]}\right)$$
$$+V_1\cdot((Kss\cdot[GSH]\cdot[CS]-Kss\cdot[GS]\cdot[CSH]))$$

$$\frac{d([CS]\cdot V_1)}{dt} = -V_1\cdot((Kss\cdot[CS]\cdot[HSR]-Kss\cdot[CSH]\cdot[SR]))$$
$$+2\cdot V_1\cdot(Kox\cdot[CSH]\cdot[TbOOH])$$
$$+2\cdot V_1\cdot\left(\frac{[CSH]\cdot k1_{(Cat3a)}\cdot[Se]\cdot[TbOOH]}{k2_{(Cat3a)}\cdot([CS]+[GS]+[SR])+k3_{(Cat3a)}\cdot[TbOOH]+[CSH]+[HSR]+[GSH]}\right)$$
$$-V_1\cdot((Kss\cdot[GSH]\cdot[CS]-Kss\cdot[GS]\cdot[CSH]))$$
$$+V_1\cdot(FvsV\cdot CS\_In)$$
$$-V_1\cdot(FvsV\cdot[CS])$$

$$\frac{d([HSR]\cdot V_1)}{dt} = -V_1\cdot((Kss\cdot[CS]\cdot[HSR]-Kss\cdot[CSH]\cdot[SR]))$$
$$+V_1\cdot(KL\cdot[ThioU]\cdot[CSH])$$
$$+V_1\cdot(FvsV\cdot HSR\_In)$$
$$-2\cdot V_1\cdot(Kox\cdot[HSR]\cdot[TbOOH])$$
$$+V_1\cdot(Kh\cdot[ThioU])$$
$$+V_1\cdot((Kss\cdot[GSH]\cdot[SR]-Kss\cdot[GS]\cdot[HSR]))$$
$$-V_1\cdot(Kact\cdot[ProC]\cdot[HSR])$$
$$-2\cdot V_1\cdot\left(\frac{[HSR]\cdot k1_{(Cat3)}\cdot[Se]\cdot[TbOOH]}{k2_{(Cat3)}\cdot([CS]+[SR]+[GS])+k3_{(Cat3)}\cdot[TbOOH]+[HSR]+[CSH]+[GSH]}\right)$$
$$-V_1\cdot(FvsV\cdot[HSR])$$

$$\frac{d([SR]\cdot V_1)}{dt} = +V_1\cdot((Kss\cdot[CS]\cdot[HSR]-Kss\cdot[CSH]\cdot[SR]))$$
$$+2\cdot V_1\cdot(Kox\cdot[HSR]\cdot[TbOOH])$$
$$-V_1\cdot((Kss\cdot[GSH]\cdot[SR]-Kss\cdot[GS]\cdot[HSR]))$$
$$+2\cdot V_1\cdot\left(\frac{[HSR]\cdot k1_{(Cat3)}\cdot[Se]\cdot[TbOOH]}{k2_{(Cat3)}\cdot([CS]+[SR]+[GS])+k3_{(Cat3)}\cdot[TbOOH]+[HSR]+[CSH]+[GSH]}\right)$$
$$-V_1\cdot(FvsV\cdot[SR])$$

$$\frac{d([GSH]\cdot V_1)}{dt} = +V_1\cdot(KL\cdot[ThioU]\cdot[CSH])$$
$$-V_1\cdot(FvsV\cdot[GSH])$$
$$-2\cdot V_1\cdot(Kox\cdot[GSH]\cdot[TbOOH])$$
$$-V_1\cdot((Kss\cdot[GSH]\cdot[SR]-Kss\cdot[GS]\cdot[HSR]))$$
$$-V_1\cdot(Kact\cdot[ProC]\cdot[GSH])$$
$$-2\cdot V_1\cdot\left(\frac{[GSH]\cdot k1_{(Cat3b)}\cdot[Se]\cdot[TbOOH]}{k2_{(Cat3b)}\cdot([CS]+[SR]+[GS])+k3_{(Cat3b)}\cdot[TbOOH]+[GSH]+[CSH]+[HSR]}\right)$$
$$-V_1\cdot((Kss\cdot[GSH]\cdot[CS]-Kss\cdot[GS]\cdot[CSH]))$$

$$\frac{d([ThioU]\cdot V_1)}{dt} = -V_1\cdot(KL\cdot[ThioU]\cdot[CSH])$$
$$-V_1\cdot(Kh\cdot[ThioU])$$
$$+V_1\cdot(FvsV\cdot ThioU\_In)$$
$$-V_1\cdot(FvsV\cdot[ThioU])$$

$$\frac{d([GS]\cdot V_1)}{dt} = -V_1\cdot(FvsV\cdot[GS])$$
$$+2\cdot V_1\cdot(Kox\cdot[GSH]\cdot[TbOOH])$$
$$+V_1\cdot((Kss\cdot[GSH]\cdot[SR]-Kss\cdot[GS]\cdot[HSR]))$$
$$+2\cdot V_1\cdot\left(\frac{[GSH]\cdot k1_{(Cat3b)}\cdot[Se]\cdot[TbOOH]}{k2_{(Cat3b)}\cdot([CS]+[SR]+[GS])+k3_{(Cat3b)}\cdot[TbOOH]+[GSH]+[CSH]+[HSR]}\right)$$
$$+V_1\cdot((Kss\cdot[GSH]\cdot[CS]-Kss\cdot[GS]\cdot[CSH]))$$

$$\frac{d([TbOOH]\cdot V_1)}{dt} = +V_1\cdot(FvsV\cdot TbOOH\_in)$$
$$-V_1\cdot(FvsV\cdot[TbOOH])$$
$$-V_1\cdot(Kox\cdot[HSR]\cdot[TbOOH])$$
$$-V_1\cdot(Kox\cdot[GSH]\cdot[TbOOH])$$
$$-V_1\cdot(Kox\cdot[CSH]\cdot[TbOOH])$$
$$-V_1\cdot\left(\frac{[HSR]\cdot k1_{(Cat3)}\cdot[Se]\cdot[TbOOH]}{k2_{(Cat3)}\cdot([CS]+[SR]+[GS])+k3_{(Cat3)}\cdot[TbOOH]+[HSR]+[CSH]+[GSH]}\right)$$
$$-V_1\cdot\left(\frac{[CSH]\cdot k1_{(Cat3a)}\cdot[Se]\cdot[TbOOH]}{k2_{(Cat3a)}\cdot([CS]+[GS]+[SR])+k3_{(Cat3a)}\cdot[TbOOH]+[CSH]+[HSR]+[GSH]}\right)$$
$$-V_1\cdot\left(\frac{[GSH]\cdot k1_{(Cat3b)}\cdot[Se]\cdot[TbOOH]}{k2_{(Cat3b)}\cdot([CS]+[SR]+[GS])+k3_{(Cat3b)}\cdot[TbOOH]+[GSH]+[CSH]+[HSR]}\right)$$

$$\frac{d([ProC]\cdot V_1)}{dt} = -V_1\cdot(Kact\cdot[ProC]\cdot[HSR])$$
$$-V_1\cdot(Kact\cdot[ProC]\cdot[CSH])$$
$$-V_1\cdot(Kact\cdot[ProC]\cdot[GSH])$$
$$+V_1\cdot(FvsV\cdot ProC\_in)$$
$$-V_1\cdot(FvsV\cdot[ProC])$$

$$\frac{d([Se]\cdot V_1)}{dt} = +V_1\cdot(Kact\cdot[ProC]\cdot[HSR])$$
$$+V_1\cdot(Kact\cdot[ProC]\cdot[CSH])$$
$$+V_1\cdot(Kact\cdot[ProC]\cdot[GSH])$$
$$-V_1\cdot(FvsV\cdot[Se])$$
$$+V_1\cdot(FvsV\cdot Se\_in)$$

Supplementary Figure 18. The ODEs automatically generated by COPASI. V_1 can be ignored because the experiment involves only one compartment.

These ODEs can be used to model both oscillators with catalyst **2** or with pre-catalysts **3**-**5**. In the model with catalyst **2**, the input concentration of pre-catalyst (ProC_in) is set to 0, whereas Se_in is set to 2·[**2**] in the incoming flow. In the model with pre-catalysts **3**-**5**, Se_in is

set to 0, whereas the input concentration of pre-catalyst (ProC_in) is set to the desirable value.

The parameters used in all models were set as follows: $k_{SS} = 2\ M^{-1}s^{-1}$, $k_H = 5\times10^{-5}\ s^{-1}$, $k_L = 2.5\ M^{-1}s^{-1}$, $k_{Ox} = 0.07\ M^{-1}s^{-1}$. The k_1, k_2, and k_3 values correspond to k_{cat}, k_{inh}, and k_{OOH}, and were individually defined for each thiol; for thiocholine: $k_1 = 9\ M^{-1}s^{-1}$, $k_2 = 0.0025$, and $k_3 = 0.006$. For cysteamine and 2-mercaptoethylguanidine: $k_1 = 9\ M^{-1}s^{-1}$, $k_2 = 0.036$, and $k_3 = 0.08$. k_{Act} was 1.75 $M^{-1}s^{-1}$, 1.14 $M^{-1}s^{-1}$, and 0.33 $M^{-1}s^{-1}$ for **3**, **4**, and **5** correspondingly. The concentration of reagents in the incoming flow and flow/V values were taken as in the experiments (Figure 5 main text)

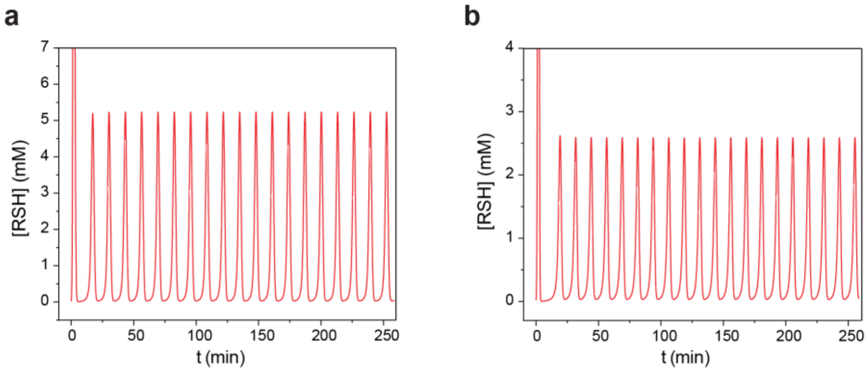

Supplementary Figure 19. Modeling of selenium catalyzed oxidation-based oscillators. **a**. Simulations of the oscillations with catalyst **3**. The concentrations of reagents and the flow rate are the same as in the experiment shown in Figure 5b. **b**. Simulations of the oscillations with catalyst **4**. The concentrations of reagents and the flow rate are the same as in the experiment shown in Figure 4c.

7. **NMR spectra**

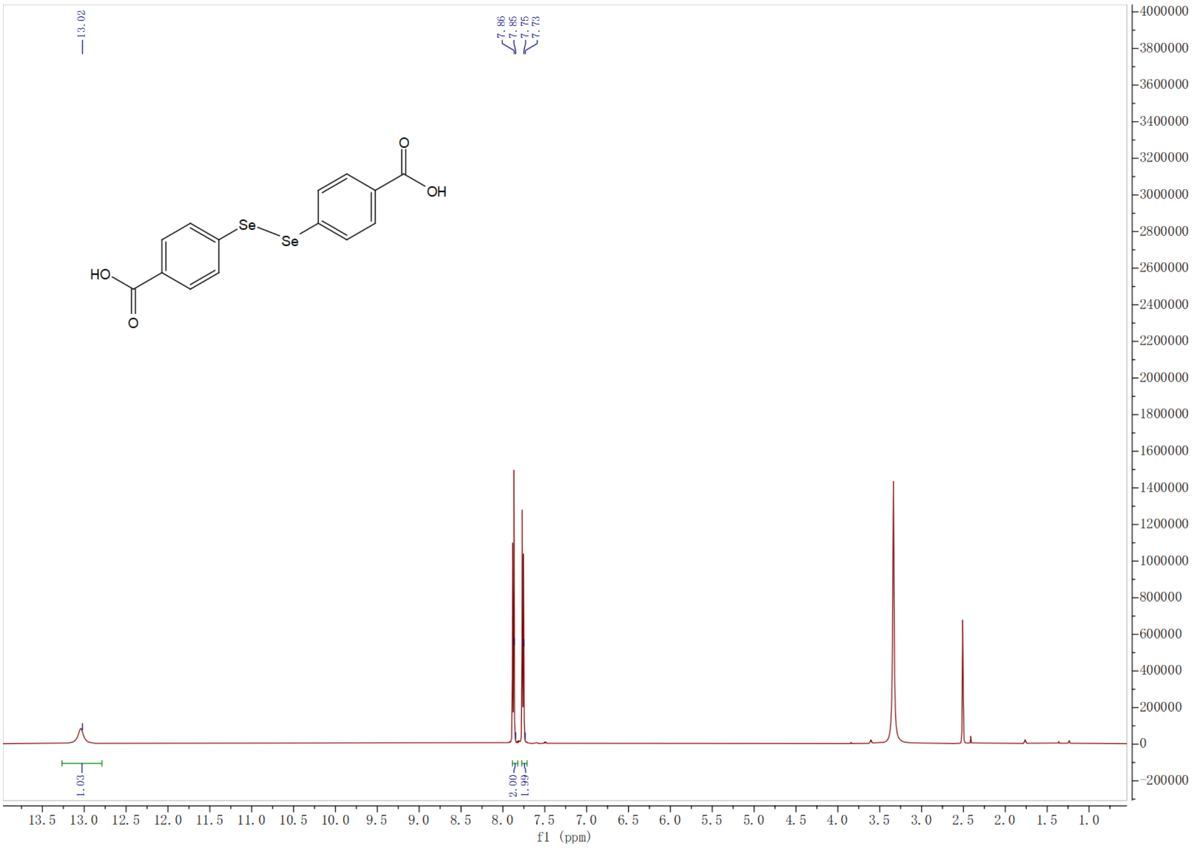

Supplementary Figure 20. ^{1}H NMR of catalyst **2** (500 MHz, $CDCl_3$).

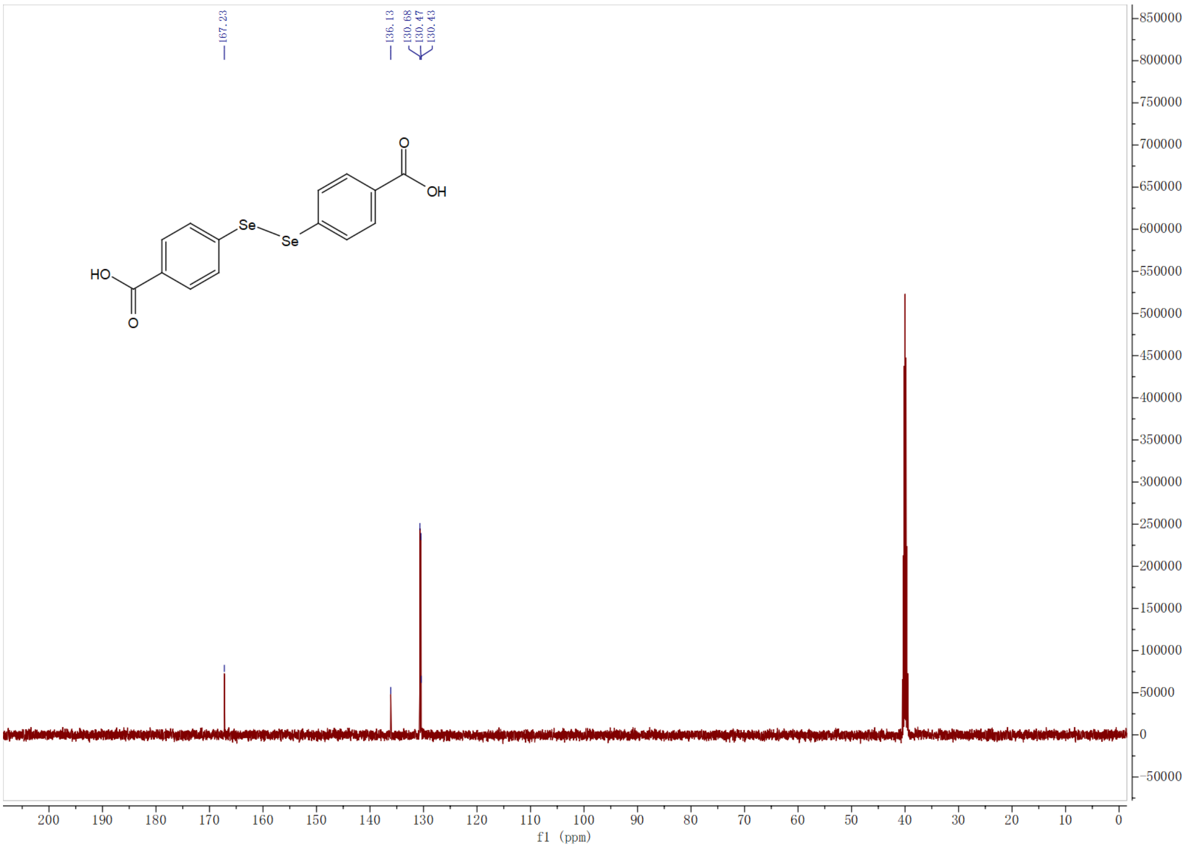

Supplementary Figure 21. ^{13}C NMR of catalyst **2** (126 MHz, $CDCl_3$).

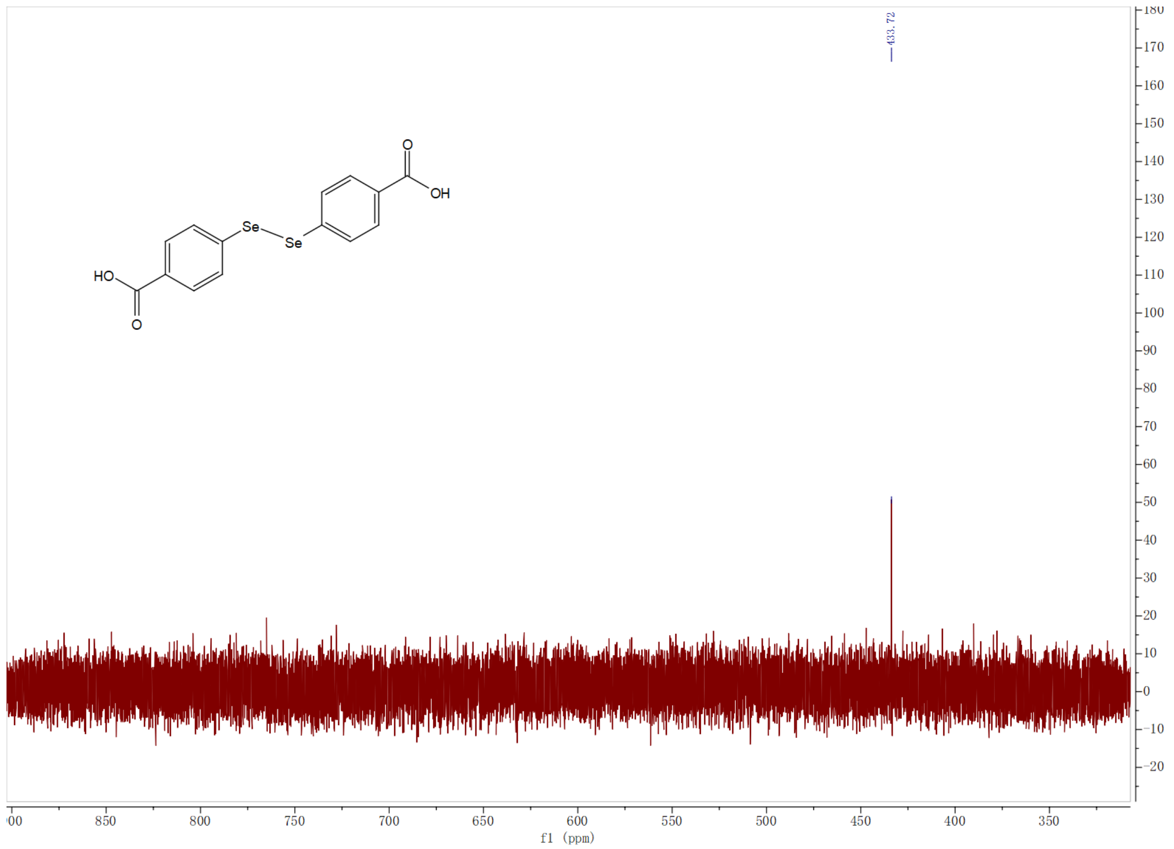

Supplementary Figure 22. ^{77}Se NMR of catalyst **2** (95 MHz, $CDCl_3$).

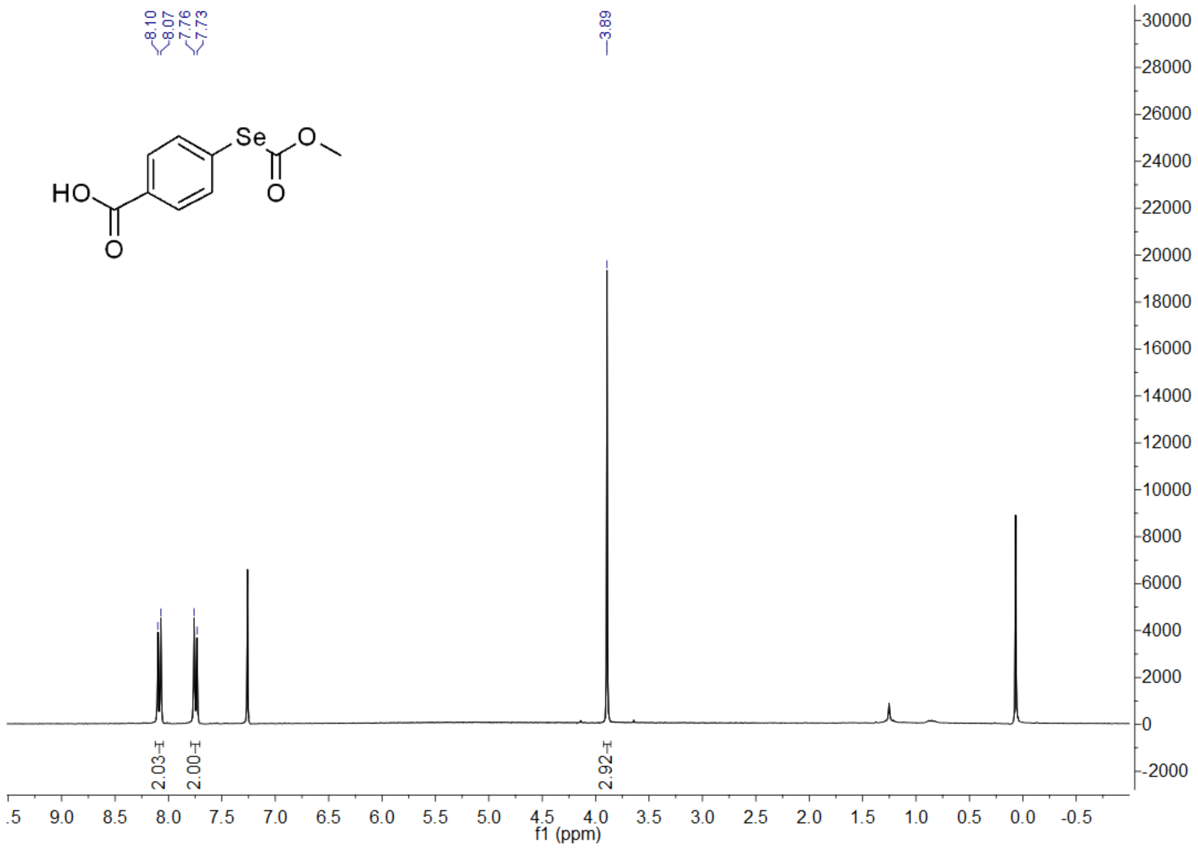

Supplementary Figure 23. ^{1}H NMR of catalyst **3** (300 MHz, $CDCl_3$).

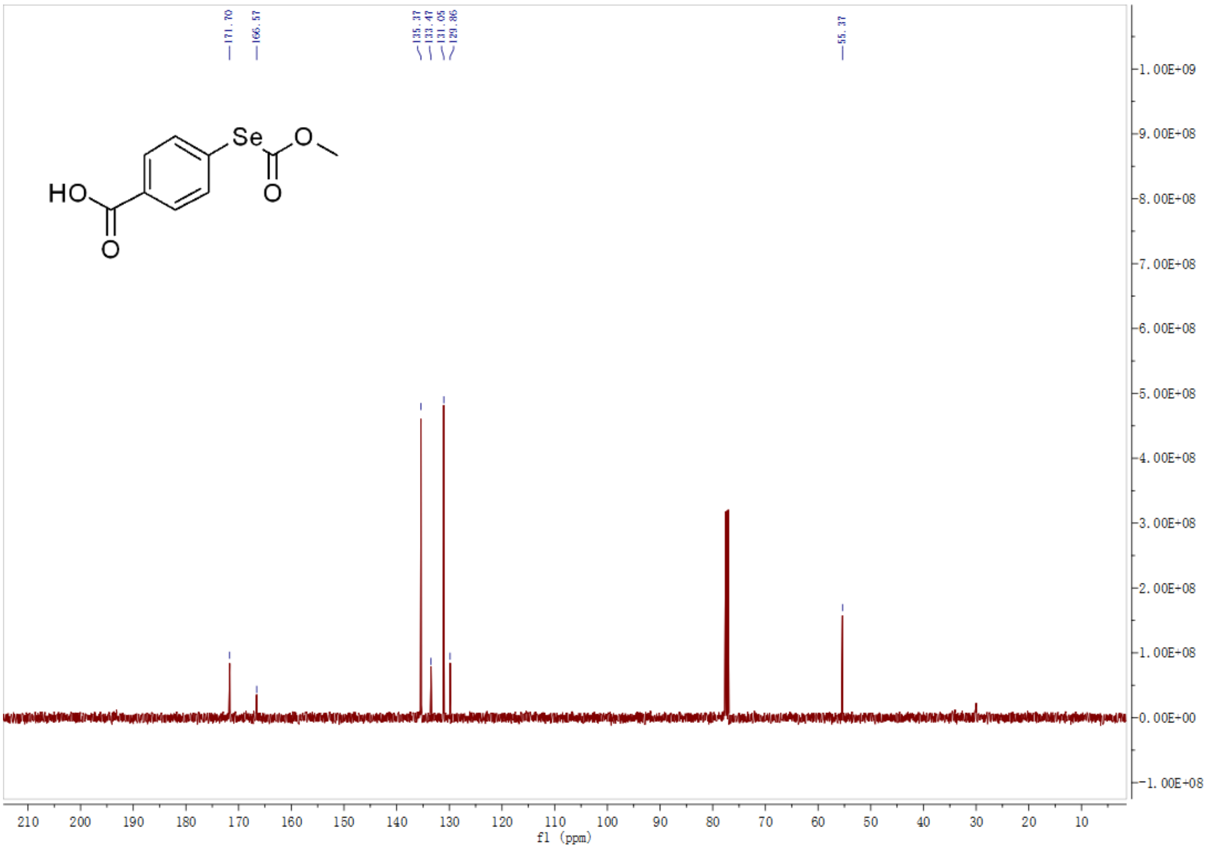

Supplementary Figure 24.^{13}C NMR of catalyst **3** (126 MHz, $CDCl_3$).

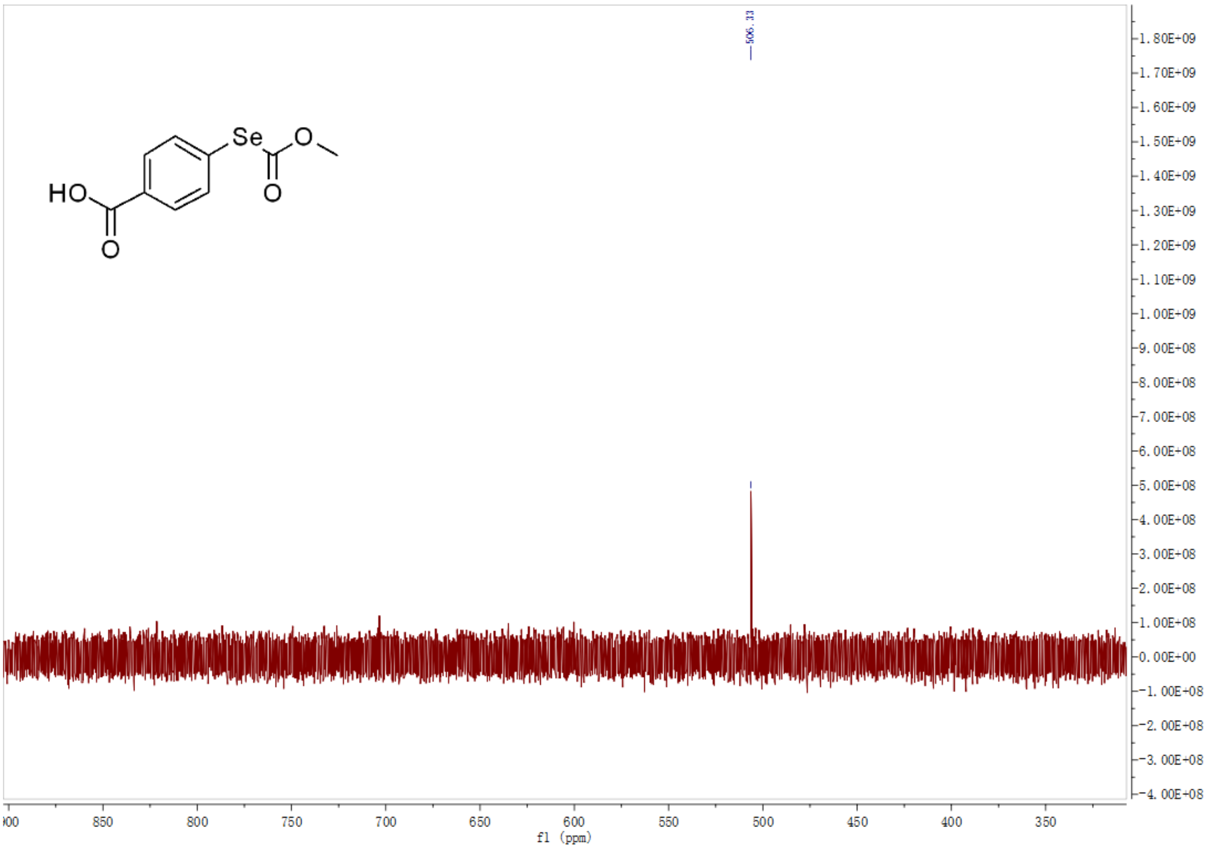

Supplementary Figure 25.^{77}Se NMR of catalyst **3** (95 MHz, $CDCl_3$).

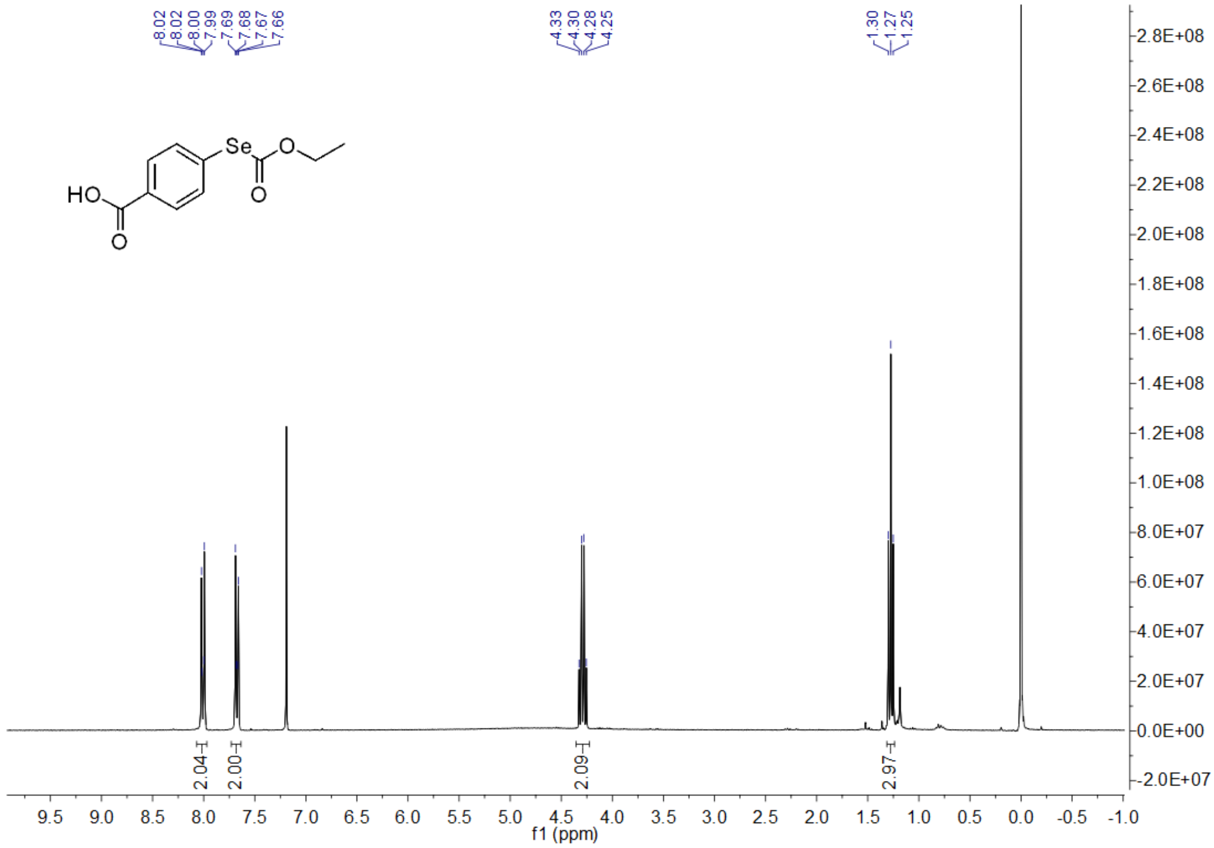

Supplementary Figure 26. ^{1}H NMR of catalyst **4** (300 MHz, $CDCl_3$).

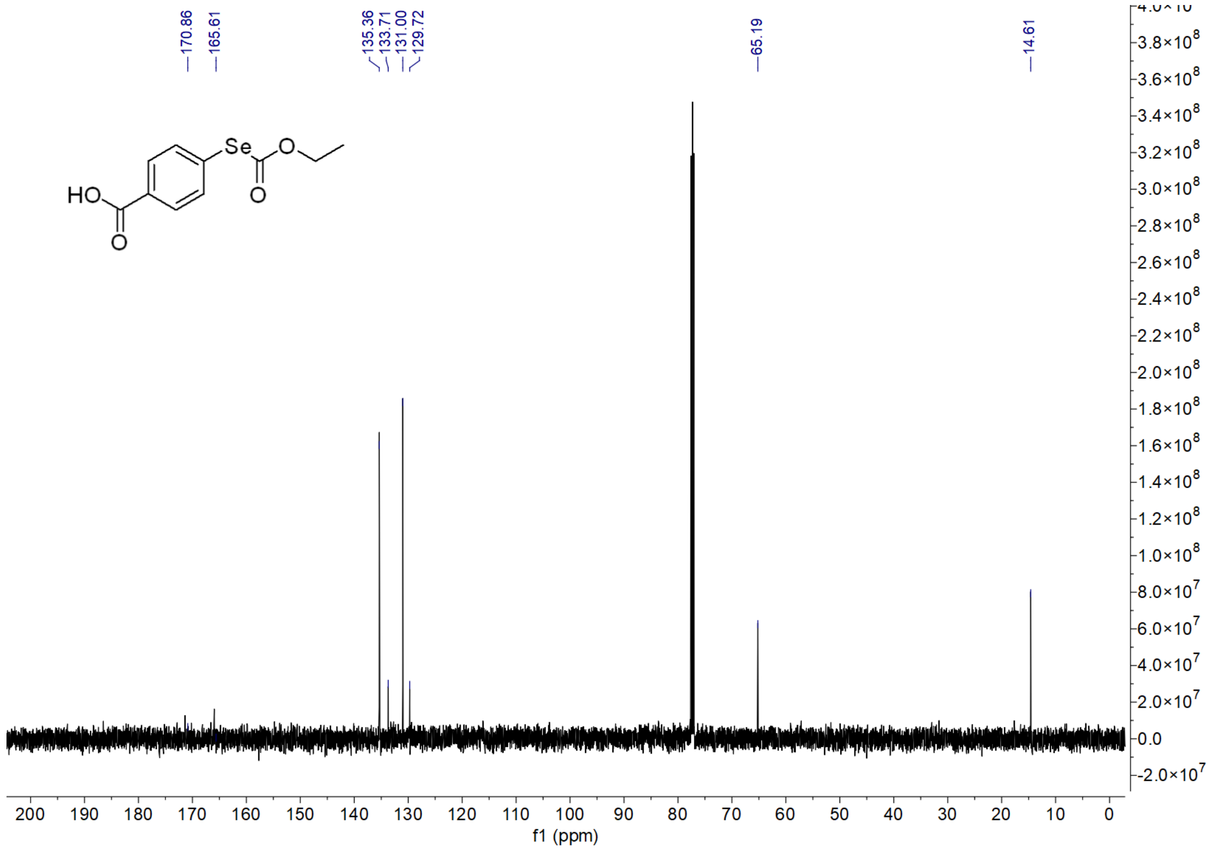

Supplementary Figure 27.^{13}C NMR of catalyst **4** (126 MHz, $CDCl_3$).

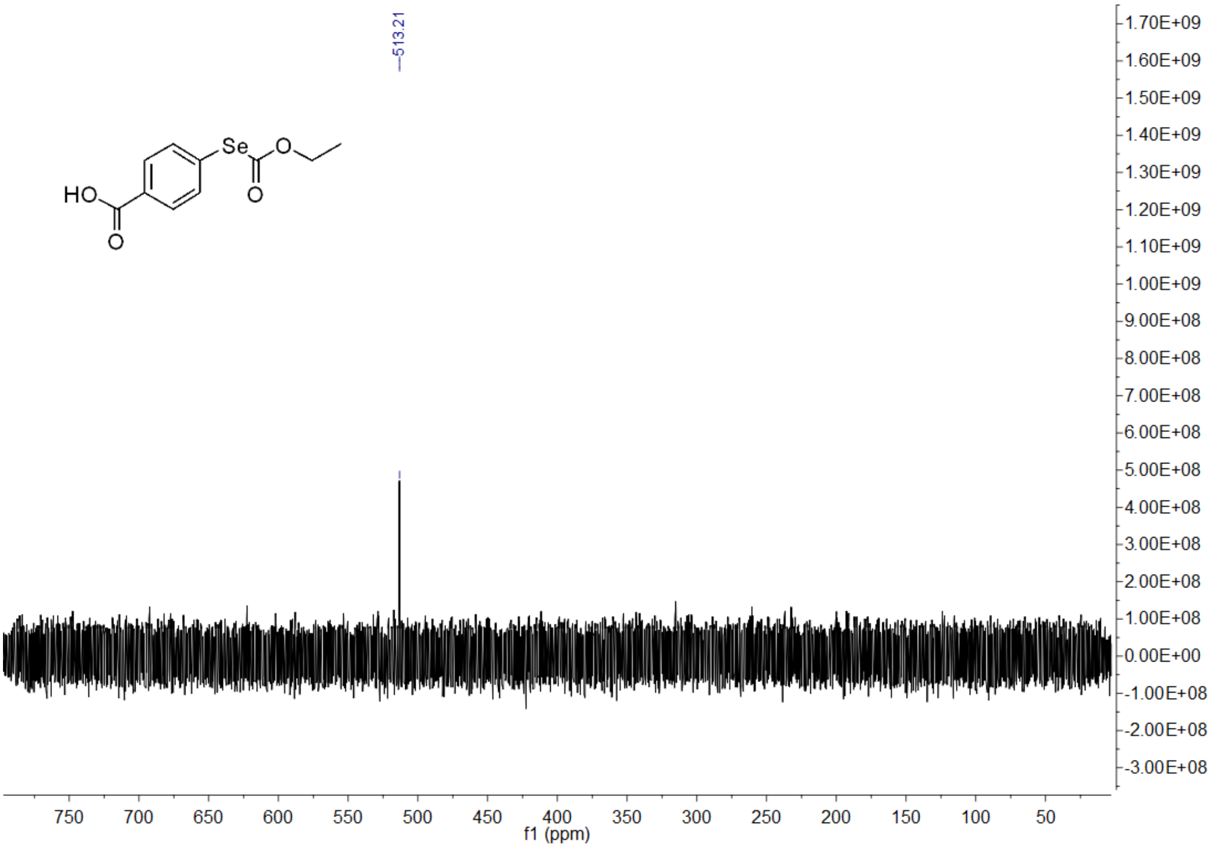

Supplementary Figure 28. ^{77}Se NMR of catalyst **4** (95 MHz, $CDCl_3$).

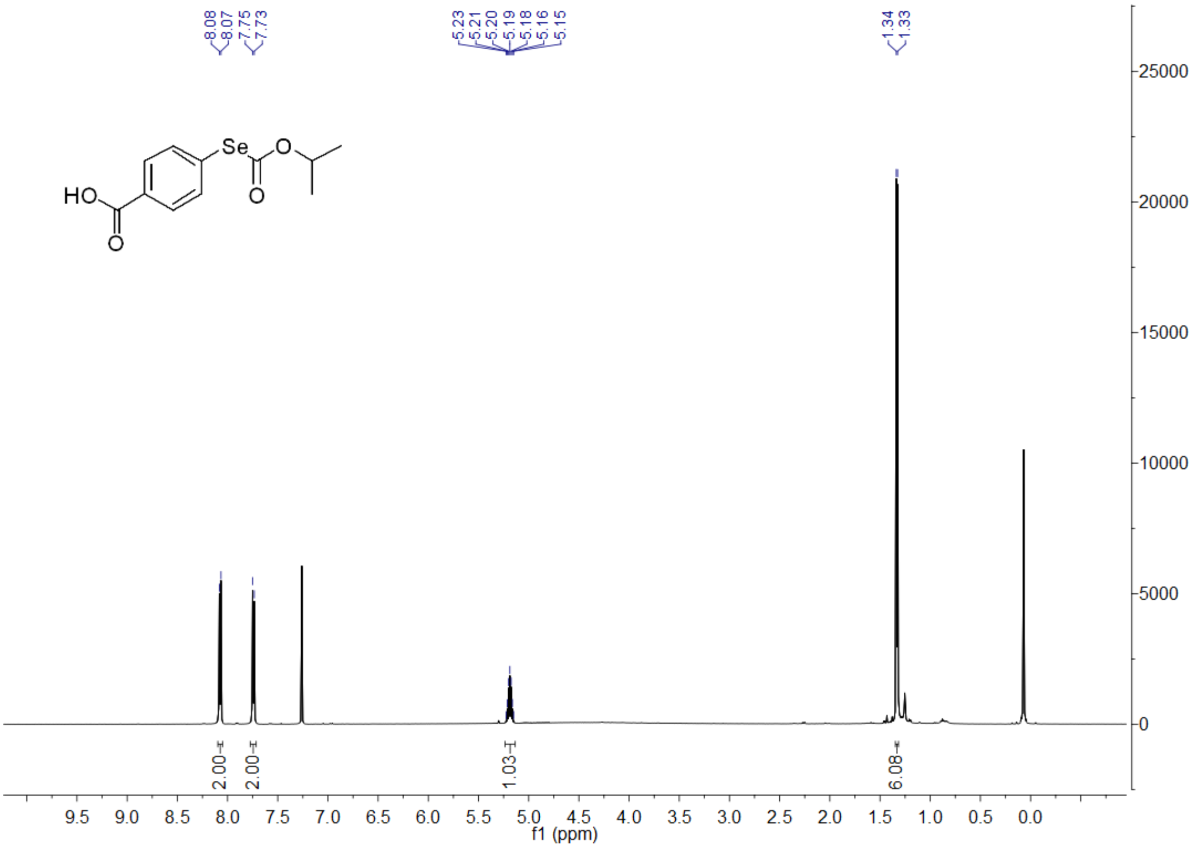

Supplementary Figure 29. ^{1}H NMR of catalyst **5** (500 MHz, $CDCl_3$).

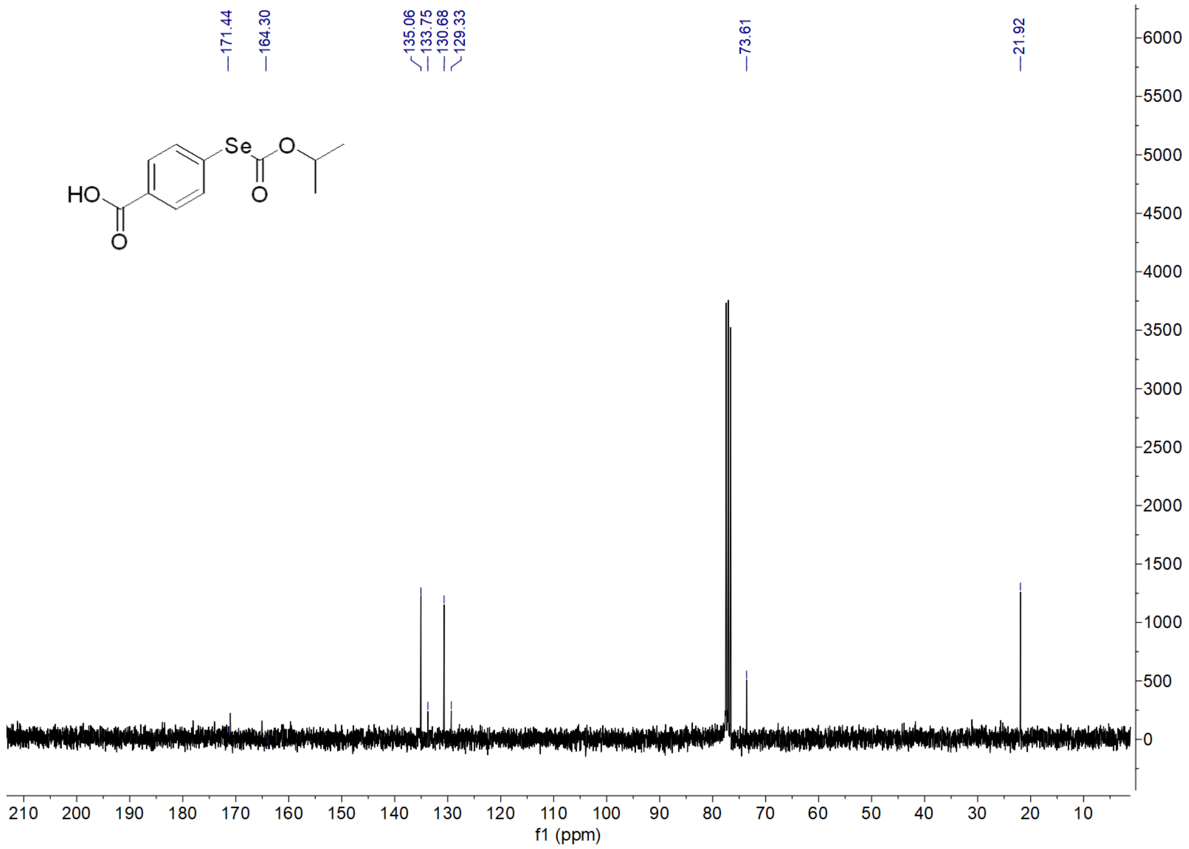

Supplementary Figure 30. ^{13}C NMR of catalyst **5** (126 MHz, $CDCl_3$).

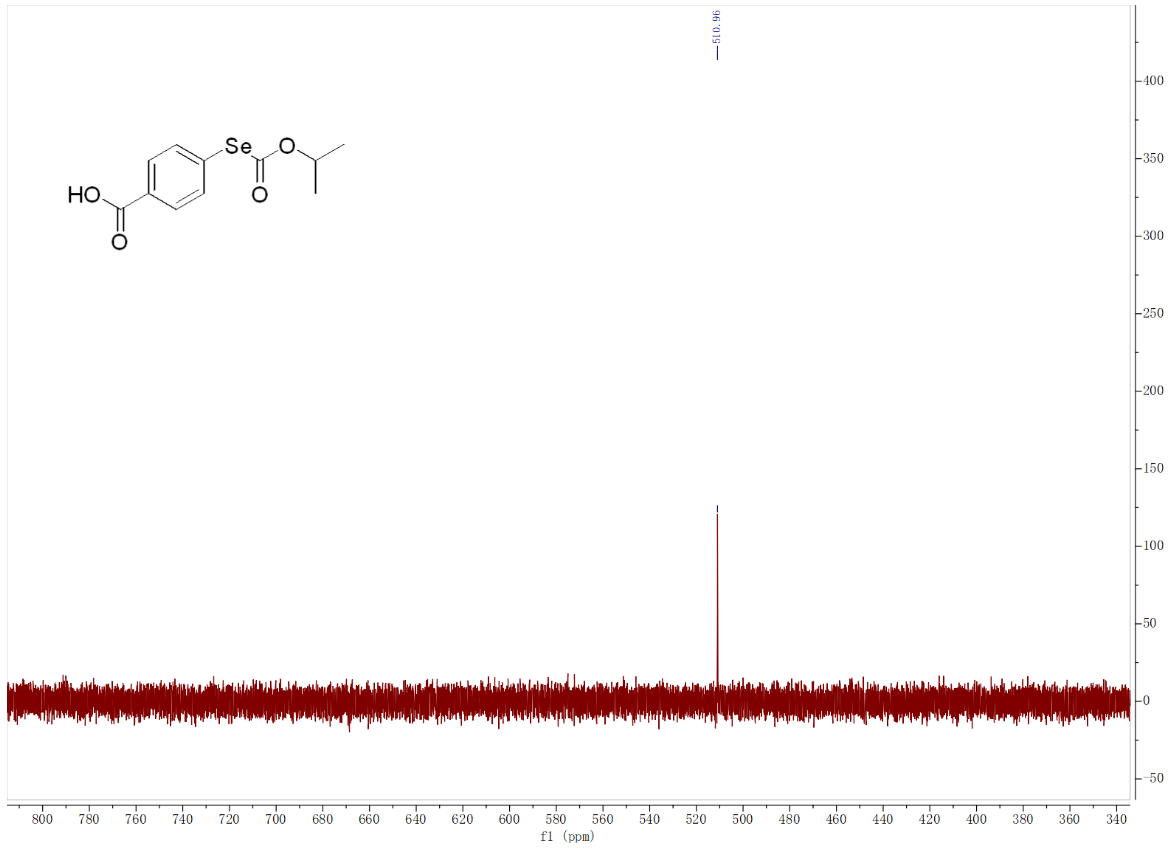

Supplementary Figure 31. ^{77}Se NMR of catalyst **5** (95 MHz, $CDCl_3$).

8. Supplementary references

1 Nakamura, K. *et al.* New selenyl linker for solid-phase synthesis of dehydropeptides. *Tetrahedron Lett.* **44**, 5445-5448 (2003).

2 Krief, A., Dumont, W. & Delmotte, C. Reaction of Organic Selenocyanates with Hydroxides: The One-Pot Synthesis of Dialkyl Diselenides from Alkyl Bromides. *Angew. Chem. Int. Ed.* **39**, 1669-1672 (2000).

3 Novichkov, A. I. *et al.* Autocatalytic and oscillatory reaction networks that form guanidines and products of their cyclization. *Nat. Commun.* **12**, 2994 (2021).

4 Pleasants, J. C., Guo, W. & Rabenstein, D. L. A comparative study of the kinetics of selenol/diselenide and thiol/disulfide exchange reactions. *J. Am. Chem. Soc.* **111**, 6553-6558 (2002).

5 Steinmann, D., Nauser, T. & Koppenol, W. H. Selenium and sulfur in exchange reactions: a comparative study. *J. Org. Chem.* **75**, 6696-6699 (2010).

6 Hoops, S. *et al.* COPASI- A COmplex PAthway SImulator. *Bioinformatics* **22**, 3067-3074 (2006).
